# Adaptive evaluation of mHealth and conventional adherence support interventions to optimize outcomes with new treatment regimens for drug-resistant tuberculosis and HIV in South Africa (ADAP-TIV): study protocol for an adaptive randomized controlled trial

**DOI:** 10.1186/s13063-023-07520-9

**Published:** 2023-12-01

**Authors:** Jesse Ross, Rubeshan Perumal, Allison Wolf, Mbali Zulu, Kevin Guzman, Boitumelo Seepamore, Karl Reis, Hlengiwe Nyilana, Senzo Hlathi, Radhamoney Narasimmulu, Ying Kuen K. Cheung, K. Rivet Amico, Gerald Friedland, Amrita Daftary, Jennifer R. Zelnick, Kogieleum Naidoo, Max R. O’Donnell

**Affiliations:** 1 Division of Pulmonary, Allergy, and Critical Care Medicine, Columbia University Irving Medical Center, Suite E101, 8th Floor, PH Building, 622 W. 168th Street, New York City, NY 10032 USA; 2 CAPRISA MRC- HIV-TB Pathogenesis and Treatment Research Unit, Durban, South Africa; 3 School of Applied Sciences, University of KwaZulu-Natal, Durban, South Africa; 4 Vagelos College of Physicians and Surgeons, Columbia University, New York City, NY USA; 5 Department of Biostatistics, Columbia University Irving Medical Center, New York City, NY USA; 6 University of Michigan School of Public Health, Ann Arbor, MI USA; 7 Yale School of Medicine, New Haven, CT USA; 8 Dahdaleh Institute of Global Health Research, School of Global Health, York University, Toronto, Canada; 9 Graduate School of Social Work, Touro University, New York City, NY USA; 10 Department of Epidemiology, Columbia University Irving Medical Center, New York City, NY USA

**Keywords:** Tuberculosis, MDR-TB, HIV/AIDS, Bedaquiline, Antiretrovirals, mHealth, South Africa, Adherence support

## Abstract

**Background:**

Highly effective, short-course, bedaquiline-containing treatment regimens for multidrug-resistant tuberculosis (MDR-TB) and integrase strand transfer inhibitor (INSTI)-containing fixed dose combination antiretroviral therapy (ART) have radically transformed treatment for MDR-TB and HIV. However, without advances in adherence support, we may not realize the full potential of these therapeutics. The primary objective of this study is to compare the effect of adherence support interventions on clinical and biological endpoints using an adaptive randomized platform.

**Methods:**

This is a prospective, adaptive, randomized controlled trial comparing the effectiveness of four adherence support strategies on a composite clinical outcome in adults with MDR-TB and HIV initiating bedaquiline-containing MDR-TB treatment regimens and receiving ART in KwaZulu-Natal, South Africa. Trial arms include (1) enhanced standard of care, (2) psychosocial support, (3) mHealth using cellular-enabled electronic dose monitoring, and (4) combined mHealth and psychosocial support. The level of support will be titrated using a differentiated service delivery (DSD)-informed assessment of treatment support needs. The composite primary outcome will include survival, negative TB culture, retention in care, and undetectable HIV viral load at month 12. Secondary outcomes will include individual components of the primary outcome and quantitative evaluation of adherence on TB and HIV treatment outcomes.

**Discussion:**

This trial will evaluate the contribution of different modes of adherence support on MDR-TB and HIV outcomes with WHO-recommended all-oral MDR-TB regimens and ART in a high-burden operational setting. We will also assess the utility of a DSD framework to pragmatically adjust levels of MDR-TB and HIV treatment support.

**Trial registration:**

ClinicalTrials.gov NCT05633056. Registered on 1 December 2022

**Supplementary Information:**

The online version contains supplementary material available at 10.1186/s13063-023-07520-9.

## Background

Tuberculosis (TB) is the second leading cause of death, after severe acute respiratory syndrome coronavirus 2 (SARS-CoV-2), due to an infectious agent and remains the leading cause of death for persons living with HIV/AIDS [1]. Multi-drug resistant tuberculosis (MDR-TB) is defined as TB resistant to rifampicin and isoniazid, the two most potent bactericidal first-line antimycobacterial drugs [1]. In 2021, there were an estimated 450,000 incident cases of people with MDR-TB globally, a 3.1% increase from 2020, with an estimated 191,000 associated deaths [1]. The 2020 global milestones to reduce TB incidence by 20% and mortality by 35% were not achieved [1, 2]; an important reason for this failure was an increase in the overall burden of MDR-TB.

Until recently, treating MDR-TB has required complex regimens with long duration and severe adverse events [3]. Despite this, MDR-TB patients experienced low rates of treatment success and high attributable mortality [1, 4]. In 2012, bedaquiline, an oral agent for MDR-TB with a novel mechanism of action, was licensed by the FDA, allowing for development of all-oral regimens. In 2020, the World Health Organization (WHO) recommended new shortened bedaquiline-containing MDR-TB regimens, following evidence of improved treatment success [3, 5]. An expanding body of evidence describes improved survival and lower treatment burden with oral short course MDR-TB regimens [6].

Among incident cases of TB, approximately 710,000, or 6.7%, were people living with HIV [1]. In South Africa, the majority (53%) of TB patients are HIV co-infected [1, 7]. Advances in antiretroviral therapy (ART) regimens utilizing second-generation integrase strand inhibitors (INSTIs) have led to enhanced HIV viral suppression, reduced acquired ART resistance, and improved clinical outcomes in low- and middle-income countries (LMICs) [8, 9]. When administered as a once-daily fixed dose combination, INSTI-based ART regimens have better tolerability, adherence, efficacy, and durability with a lower incidence of adverse events compared to older ART regimens [10–12]. Due to these positive characteristics and flexibility, INSTI-based ART regimens have been proposed as a “universal ART” for widespread use, including in patients with MDR-TB HIV co-infection [13, 14].

Differentiated service delivery (DSD) is an innovative patient-centered care model, developed for people living with HIV, tailored to their health status and clinical needs, and informed by social, behavioral, and structural factors. DSD is an approach to delivering HIV care and treatment that takes into account the diverse needs and preferences of people living with HIV, and seeks to provide high-quality care responsive to those needs [15–17]. DSD modalities may include mHealth, psychosocial support, adherence support groups, and community-based care. In South Africa, our team has piloted mHealth-guided adherence support using electronic dose monitoring, adherence support groups, and individual counseling using a motivational-interviewing approach for patients co-infected with MDR-TB and HIV [17–19].

### Rationale for a differentiate service delivery titrated intervention to enhance medication adherence and improve a composite treatment outcome in MDR-TB HIV co-infection

To date, TB control programs have used directly observed therapy (DOT) as the centerpiece of treatment delivery and adherence support [20], and a DSD approach has not been adapted for the DR-TB HIV care cascade. We propose extending the DSD framework to MDR-TB HIV to enhance medication adherence and retention in care to improve clinical and biological outcomes. We will implement an innovative, adaptive adherence intervention targeting reduction of barriers and enhancement of facilitators. Consistent with a DSD framework, this adaptive, randomized trial will deliver differentiated levels of service within each intervention arm depending on participant needs, with a focus on supporting patients facing more severe adherence challenges.

Building on this foundational work, we have designed an adaptive randomized controlled trial to evaluate the effect of different modes of treatment adherence support, titrated in intensity using a DSD framework, in South African patients with MDR-TB and HIV treated with bedaquiline-containing TB regimens and ART (primarily INSTI-based), on a primary composite outcome of MDR-TB and HIV treatment success.

## Methods

### Clinical trial objectives

The primary objective of this trial is to compare the effect of adherence support interventions on clinical and biological endpoints using an adaptive randomized platform. We hypothesized that in a randomized, adaptive implementation trial, the psychosocial + mHealth support arm will be associated with improvement in a composite MDR-TB and HIV clinical outcome compared to mHealth, psychosocial support, or enhanced standard of care arms.

The primary outcome will be a comparison of the percentage of participants achieving a composite of undetectable HIV viral load, MTB culture conversion, survival, and retention in care at 12 months in each arm. Secondary outcomes will include all components of the primary outcome as well as a quantitative evaluation of adherence on TB and HIV treatment response (Table 1).

**Table 1:** Trial outcomes

**Primary outcome at 12 months post-study enrollment**
Percentage achieving the following: **1)** HIV viral load undetectable AND **2)** TB sputum culture conversion^a^ AND **3)** Survival + Retention in Care^b^
**Secondary outcomes at 12 months**
1) 12 Month TB clinical outcome 2) TB sputum culture conversion^a^ 3) Time to culture conversion 4) Permanent change in TB or ART regimen 5) HIV viral load undetectable 6) Retention in care^b^ 7) All-cause mortality 8) Time to mortality 9) Barriers, enablers, and socio-behavioral pathways to adherence

^a^Sputum TB culture shows no growth for at least two consecutive months without subsequent positive TB culture

^b^Participating in TB clinic without missing two or more consecutive TB clinic appointments

### Trial design

**Fig. 1:**
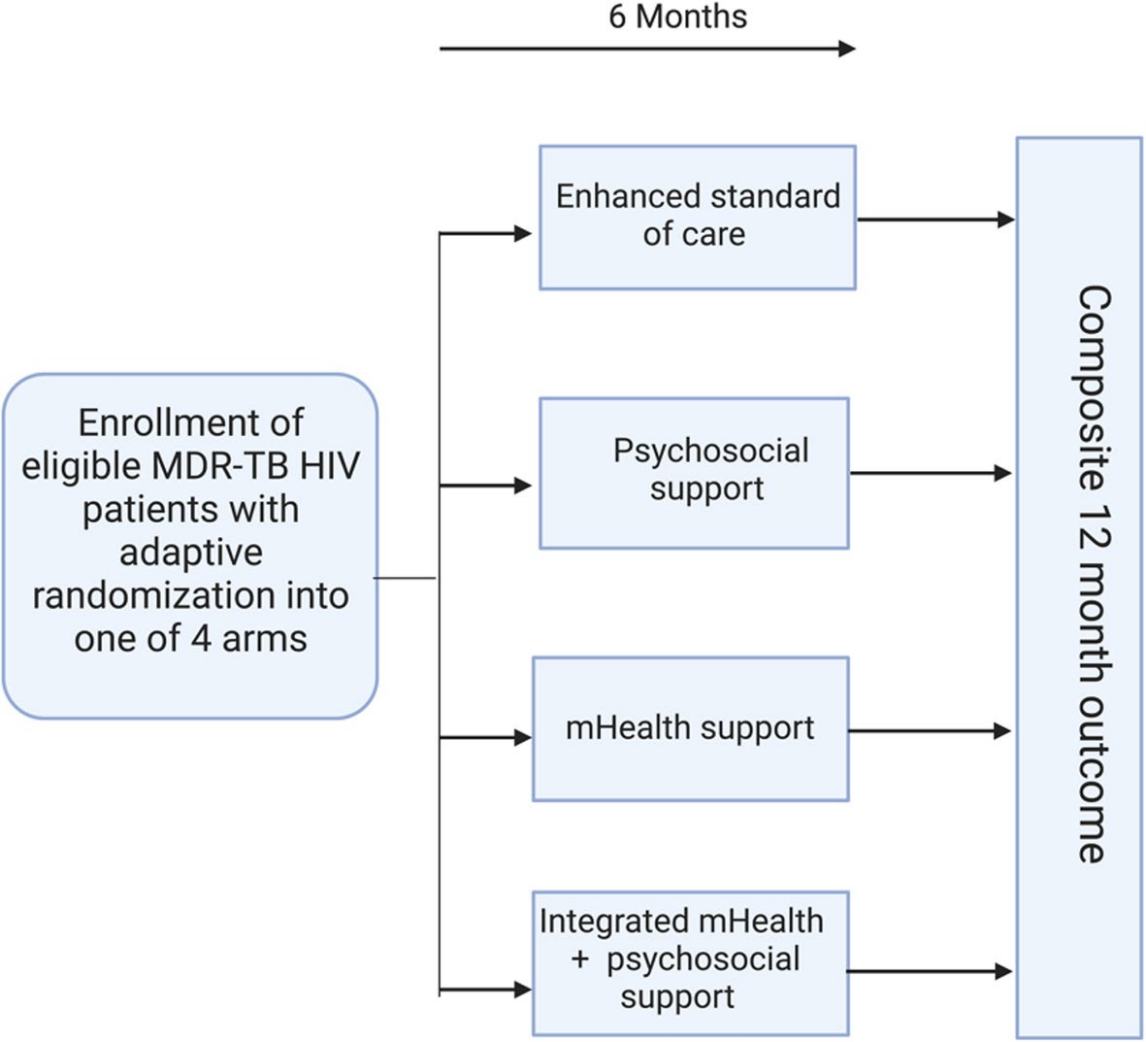
Study design overview

This study will follow a four-arm adaptive trial design to evaluate four adherence support strategies on a combined endpoint in adults with confirmed MDR-TB and HIV initiating bedaquiline-containing MDR-TB treatment regimens and on ART in KwaZulu-Natal, South Africa (Fig. 1). Interventions arms include *Arm 1* enhanced standard of care, *Arm 2* psychosocial support, *Arm 3* mHealth using cellular-enabled electronic dose monitoring, and *Arm 4* combined mHealth and psychosocial support (Table 2). The level of support will be titrated using a differentiated service delivery (DSD)-informed assessment of treatment support needs.

**Table 2:** Differentiated service delivery (DSD) elements in the four intervention arms

**Arm 1 Enhanced standard of care**
- Treatment literacy counseling
- Discharge counseling
- Monthly physician visits
- Social work and other services initiated routinely
- Study team provides extra training for physicians, nurses, and social workers to enhance standard of care
**Arm 2 Psychosocial support**
- As per Arm 1 with additional elements
- Discharge planning (if inpatient)
- Community treatment planning (if outpatient)
- Monthly counseling intervention^a^
- Community adherence support group
- Home visits^a^
**Arm 3 mHealth**
- As per Arm 1 with additional elements
- 2 Wisepill RT3000 devices; one for BDQ and one for ART
- Weekly text message^a^
- Study phone calls to support regular adherence^a^
**Arm 4 Psychosocial support + mHealth**
- As per Arm 1, 2, and 3
- Active coordination between mHealth and psychosocial support staff^a^

*BDQ* Bedaquiline, *ART* Antiretroviral therapy

^a^These elements may be increased in intensity or frequency depending on empirically assessed patient needs using a DSD framework within each Arm

### Trial population and setting

Eligible participants will be consecutively recruited adult patients (age ≥ 18 years) meeting all of the following inclusion criteria: (1) culture or molecular test positive for TB; (2) molecular test positive for HIV or with a known or documented history of HIV; (3) drug-susceptibility testing by molecular (i.e., GeneXpert MTB/RIF) or conventional testing demonstrating at least rifampicin-resistant TB; (4) initiating treatment with a bedaquiline-containing TB regimen within 4 weeks of enrollment; (5) on treatment with ART regimen, including dolutegravir-containing combination ART regimen, or starting ART within 4 weeks of enrollment; (6) capacity for informed consent in either isiZulu or English. Patients will be excluded from the study if they do not meet eligibility criteria, are prisoners, are pregnant at time of enrollment, or are considered by study team to be too ill to participate in the trial.

Participants will be recruited from MDR-TB treatment facilities and affiliated outpatient TB clinics in Durban, South Africa. Study visits will be conducted within the established research infrastructure at the Durban CAPRISA Springfield Clinical Research Site.

### Adaptive randomization

Eligible participants will be assigned to one of four intervention arms at baseline using a two-step randomization. Participants will be initially randomized to Arm 1 vs Arms 2–4 in a 1:3 ratio, with randomization stratified by baseline variables (undetectable vs detectable HIV viral load, 6-month vs. extended MDR-TB regimen, inpatient vs outpatient, history of previous bedaquiline exposure). Bayesian adaptation will be accomplished by utilizing a run-in period where the first 40 participants will be randomized without any adaptation. After the first 40 participants have been randomized and achieve preliminary outcomes at 4 months, results will be incorporated in a Bayesian fashion to modify the randomization procedure for subsequent participants weighting randomization toward favorable study arms. A web-based application (R shiny 1.7.4) will be used to perform adaptive randomization in real-time.

### Trial interventions

After assignment into one of 4 arms as described above, each participant will receive an Arm specific intervention (Table 2).

Enhanced standard of care (*Arm 1*) will include usual care as administered by hospital and clinic staff, enhanced by study staff providing treatment literacy and extra training for treating physicians, nurses, pharmacists, and social workers prior to trial initiation and periodically with refresher trainings throughout the trial duration.

The psychosocial support intervention (*Arm 2*) will include discharge or community treatment planning depending on inpatient vs. outpatient treatment status, monthly individual counseling, community adherence support groups, and home visits. The intensity of the support will be calibrated based on monthly assessment by study counselors. Participants will be administered standardized TB and ART adherence assessment questionnaires monthly. Participants with a low standardized adherence score will be considered at risk for non-adherence, will have the intensity of the intervention increased, may include telephonic check-ins by study staff and increased frequency of counseling sessions from monthly to biweekly, and may have a home visit conducted by a multidisciplinary study team. Monthly adherence support groups will not be calibrated.

Participants randomized into the mHealth intervention (*Arm 3*) will receive two Wisepill RT3000 cellular-enabled electronic pill boxes (“Wisepill”): one with ART and one with bedaquiline. They will also receive training on pill box loading, charging, and storage. Each Wisepill device will be appropriately marked to avoid confusion and stigma. Pill box openings will serve as a surrogate for adherence to ART and bedaquiline, respectively. Each participant will select a text message reminder from a guided menu of choices. Participants will receive a weekly text message encouraging regular adherence. For bedaquiline, one missed, and for ART, 2 missed EDM openings within a 2-week window (not due to technical issues) will trigger an additional text message reminding the patient to take their medication. For EDM openings recorded outside the EDM dose window or continued missed doses, it will be up to the investigators’ discretion to issue additional text messages or semi-scripted study calls to support adherence. The psychosocial support + mHealth intervention (*Arm 4*) will include a combination of Arms 2 and 3. Patients will be considered at risk for non-adherence by a low adherence score as described in Arm 2 and/or missed openings recorded by Wisepill devices as described in Arm 3 and increased support will be delivered as described above.

### Trial timeline

**Fig. 2:**
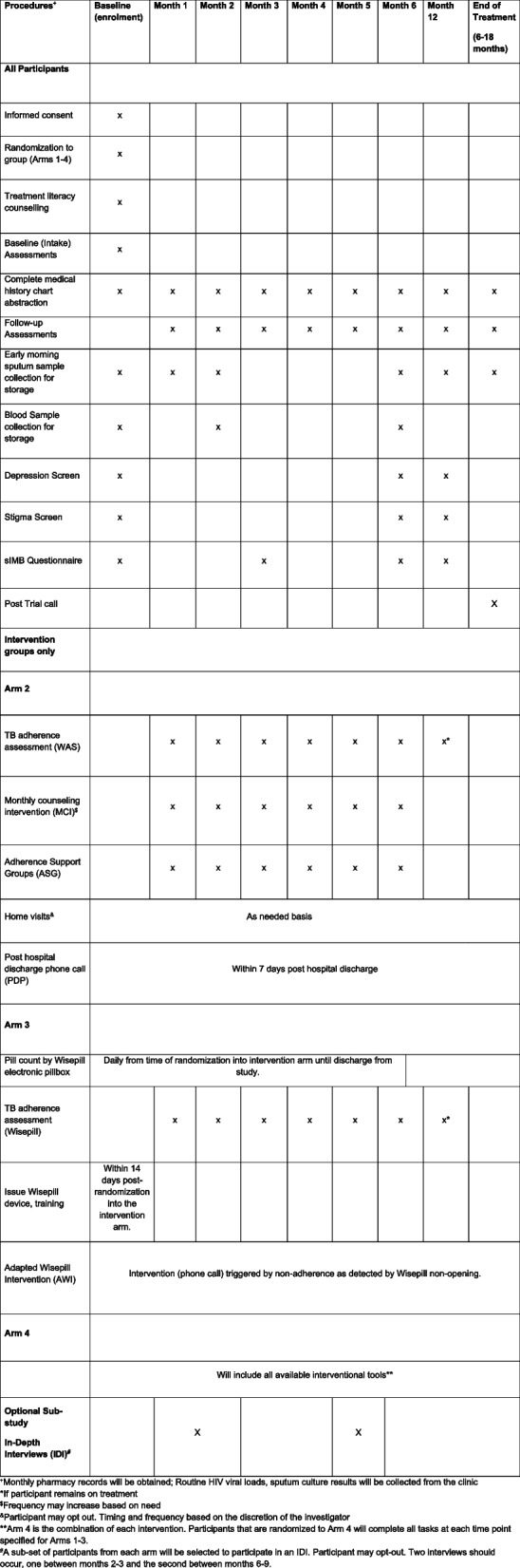
Schedule of study evaluations

The trial timeline will include 6 months to train study staff on the protocol, including the randomization strategy and software use. Approximately 4 years will be allowed for enrollment. Participants will be followed monthly through the 6 months of intervention with an additional in-person visit at 12 months to establish the primary outcome, and through the end of treatment (approximately 18 months after treatment initiation) telephonically. Data for the study will be collected at the following visits: (a) baseline (enrollment) visit, (b) monthly clinical visits (months 1–6), (c) follow-up or end of treatment visit, and (d) at community adherence support groups (Fig. 2). Sputum will be collected monthly, and serum for HIV viral load testing will be collected at months 0, 2, 6, and 12.

### Informed consent

Research assistants will recruit and enroll participants. Screening of participants is done at hospital and clinic facilities primarily at King Dinuzulu Hospital and KwaMashu community clinic. If a participant meets the protocol eligibility criteria, an informed consent procedure is conducted and the participant is then enrolled. A member of the study staff will explain the protocol and informed consent documents, with opportunity to ask questions, prior to seeking informed consent in the preferred language of the potential participant (English or isiZulu). Enrollment can occur on a different day from screening.

### Statistical analysis

The study is powered to enroll a total of 400 participants, who will be randomly assigned to one of four trial arms (Table 2). A Bayesian adaptive randomization algorithm will be used to assign interventions to participants to improve the power of detecting any superior arm(s) and to increase the number of participants treated in the better arm(s) during the study. We varied the estimated success rate, based on prior observational data in the same population, between 70 and 90% with power as low as 74% and as high as 98% depending on differences in success rates (Table 3). Family-wise error rate, the probability of erroneously declaring *any* non-superior arm(s) superior, will be between 0.005 and 0.020 depending on treatment success in the various arms.

**Table 3:** Adaptive study design power analysis showing two possible effect estimates and various scenarios

**Scenario**	**Success rate (%)**				**Power**^**a**^	**FWER**^**b**^
	Standard	mHealth	Socio-behavioral	mHealth + socio-behavioral		
Null	**70%**	70%	70%	70%	---	0.019
Single	70%	70%	70%	**90%**	0.95	0.010
Two	70%	70%	**90%**	**90%**	0.97	0.006
All	70%	**90%**	**90%**	**90%**	0.98	---
Additive	70%	80%	80%	90%	0.92	---
Null	75%	75%	75%	75%	---	0.020
Single	75%	75%	75%	**90%**	0.77	0.009
Two	75%	75%	**90%**	**90%**	0.84	0.005
All	75%	**90%**	**90%**	**90%**	0.87	---
Additive	75%	**80%**	**83%**	**90%**	0.74	---

^a^*Power* Probability of correctly identifying at least one superior arm compared to standard

^b^*FWER* Family-wise error rate, the probability of erroneously declaring any non-superior arm(s) superior

### Data management

Prior to enrollment, all research staff will participate in Human Subjects Protection training/Good Clinical Practice training to ensure sensitive data confidentiality. Informed Consent Forms and all forms containing patient identifiers will be kept separate from study forms in a secure, locked location. Upon enrollment, participants will be assigned a unique study identifier (PID) assigned by the CAPRISA Data Management Center, which will be used on all case record forms (CRFs) to identify the participant for the duration of the study. RedCap software will be utilized for the development of study forms, data entry, and data management of electronic data. Electronic data will be kept securely on encrypted and password-protected end point devices with support from the CAPRISA Data Management Core. Users on the study team will have access to the study database with individual login credentials including username and password. Only designated members of the study staff will have access to the key linking the study PID data to patient identifiers.

### Data safety monitoring and review

As a single-center study, there is no coordinating center. The trial steering committee is comprised of the co-investigators of the study, abbreviated from the author list: M.O., J.Z., R.P., K.N. Meetings are occurring weekly at CAPRISA along with a steering committee meeting occurring monthly for the duration of the trial. Quality checks will be performed on the data entered into the RedCap database and completed CRFs will be checked by the quality control officers. CAPRISA data managers will verify and validate patient data and ensure Quality Control reports are produced and approved per CAPRISA data management Standard Operating Procedures (SOPs). The CAPRISA laboratory manager will ensure that all involved laboratories are compliant with Good Laboratory Practice. The CAPRISA pharmacist will provide oversight for the preparation of the electronic pillboxes and pill counts. Quality assurance/quality control of data will be undertaken according to established CAPRISA SOPs.

### Community involvement

Engagement with the provincial department of health and local health personnel has been initiated. Community engagement through the CAPRISA community program and a study community advisory board (CAB) is ongoing.

### Ethics and dissemination

The study will be conducted in compliance with South African, US, national, and local regulations and guidelines applicable to research involving human subjects, and in accordance with the International Conference on Harmonization/Good Clinical Practice. This is a minimal risk study in which investigational products or devices are not being used. Adverse events, or serious adverse events, are submitted to the University of KwaZulu-Natal (UKZN) Biomedical Research Ethics Committee (BREC) for review within standard guidelines. The trial protocol has received approval from the IRB at Columbia University and UKZN BREC. The Trial Steering Committee and CAPRISA data managers will meet to review trial conduct quarterly throughout the trial period. Protocol amendments are generated by the study team led by the PI and subsequently submitted for review to BREC (CAPRISA, South Africa) and the Columbia University Human Research Protection Office prior to implementation. The revised protocol will be maintained in the Investigator Site File and updated in the clinical trials registry. Deviations from the Protocol will be fully documented and a record kept at both CAPRISA and Columbia sites of all amendments and renewals that occur. The funder will be notified of protocol updates through protocol reporting. Results of this study will be made available and communicated to participants, healthcare professionals, and the public via publication and trial registry.

## Discussion

### Summary

Each year, approximately 14,000 people are diagnosed with MDR-TB and HIV in South Africa [1]. Medication adherence is a key predictor of TB and HIV treatment outcomes and emergent resistance to both ART and TB medications, and dual adherence to TB medications and ART remains severely understudied in high-burden TB/HIV settings [21–23]. This trial sited in Durban South Africa, global epicenter of the MDR-TB HIV syndemic, will allow us to evaluate the individual and combined contributions of mHealth and psychosocial support in the treatment of MDR-TB in people living with HIV. Using a DSD framework, we will characterize the intensity of support required to promote adherence to TB medication within each trial arm. In addition, this trial will inform optimal management of MDR-TB and HIV using cutting-edge TB and HIV treatment regimens in an operational public health system in a high-burden LMIC setting.

### Limitations

Actual and potential limitations to this trial include potential challenges to feasible implementation in a low-burden setting, lack of generalizability, potential for insufficient adherence support, and potential lack of power. Utilization of the Wisepill RT3000 device (~US $100/device) may be cost prohibitive in LMICs. However, devices may be re-used, less costly EDMs are available, and the cost of MDR-TB treatment failure is high and cascades when the cost associated with community transmission is included. We anticipate following this trial, if successful, with a cost-effectiveness analysis and potentially a larger more operational trial using a less expensive EDM. The MDR-TB HIV co-infection syndemic in KwaZulu-Natal, South Africa, is unique in its intensity as well as cultural and health systems factors. These results while immediately generalizable to similar southern African settings may not be entirely generalizable to MDR-TB treatment in Southeast Asia for example. There is a concern that the intervention arms may not be sufficiently impactful to change deep-seated behavior (“underdosed”) since adherence challenged patients may have substantial, refractory behavioral or structural challenges to adherence. While this may be accurate, a more intensive interventional approach was felt to be not feasible since it may not be implementable in routine programmatic settings. Although we consider our trial adequately powered based on assumptions derived from previous data, if the interventions perform similarly or rates of overall success are higher than expected, the trial may be unpowered to demonstrate a true difference between intervention arms.

## Conclusion

We anticipate this randomized, adaptive trial design to be a highly efficient and ethical approach to evaluating the comparative effectiveness of mHealth and psychosocial support to improve MDR-TB HIV co-infection outcomes. Furthermore, this trial will extend a DSD framework, previously developed to support HIV treatment and primarily focused on patients who are stable on ART, to patients who are co-infected with MDR-TB and HIV, including those who struggle with medication adherence. This trial takes advantage of our team’s depth of experience with this population and substantial pilot evidence to design and implement an intervention. We also use the opportunity of the roll out of bedaquline-based all-oral short course regimens for MDR-TB treatment and the availability of INSTI-based fixed dose combination ART in the South African public health system to study operational issues around MDR-TB/HIV treatment. Finally, we are in close communication with the South African public health system as we implement this trial including provincial and national laboratories and public health bodies.

## Trial status

Protocol Version 3.0 | 2FEB2023.

Start of recruitment: March 06, 2023.

Approximate completion of recruitment: March 01, 2027.

## Trial registration

ClinicalTrials.gov: NCT05633056.

Date of trial registration: December 1^st^, 2022.

### Supplementary Information

**Additional file 1.** Protocol.

**Additional file 2.** SPIRIT Checklist for Trials.

## Data Availability

Only the South Africa PI and the study team at CAPRISA will have access to the data key and identifier-containing documents. In accordance with the law, data may be reviewed by representatives of the IRB/IEC and individuals tasked with duties of monitoring and quality assurance. Any data required to support the protocol can be supplied on request.
