# Supplementary material for: Adaptive evaluation of mHealth and conventional adherence support interventions to optimize outcomes with new treatment regimens for drug-resistant tuberculosis and HIV in South Africa (ADAP-TIV): study protocol for an adaptive randomized controlled trial
Source: Trials. 2023 Dec 1;24:776. doi: 10.1186/s13063-023-07520-9 (PMC10691086; doi:10.1186/s13063-023-07520-9)
Supplement: Supplementary file 1 — Additional file 1. Protocol. [file 13063_2023_7520_MOESM1_ESM.docx]

**PROTOCOL**

Adaptive evaluation of mHealth and conventional adherence support interventions to optimize outcomes with new treatment regimens for drug-resistant tuberculosis and HIV in South Africa

Version 3.0, 29 March 2023

Sponsored by

National Institutes of Health (NIH)
Grant #: R01 AI167795-01A1

*Study Principal Investigators*

Max O’Donnell, MD, MPH, Principal Investigator (US)

Kogieleum Naidoo, MBChB, PhD Principal Investigator (South Africa)

**Acronyms**

ART Antiretroviral Therapy

AIDS Acquired Immunodeficiency Syndrome

BAR Bayesian adaptive randomization

BDQ Bedaquiline

BREC Biomedical Ethics Research Council

CAPRISA Centre for the AIDS Programme of Research in South Africa

CDC Centers for Disease Control and Prevention (United States)

CD4 CD4 lymphocyte or helper T cell (a type of white blood cell)

DR-TB Drug resistant tuberculosis

DSD Differential Service Delivery

FGD Focus Group Discussion

GCP Good Clinical Practice

HCW Health care worker

HIV Human Immunodeficiency Virus

HRPO Human Research Protection Offices

IATA International Air Transport Association

ICH International Conference on Harmonisation

IRB Institutional Review Board

KDH King DinuZulu Hospital Complex

KZN KwaZulu-Natal

MDR-TB Multi-drug resistant tuberculosis

WAS Wilson’s adherence scaleM/XDR-TB Multi-drug resistant tuberculosis or Extensively drug resistant tuberculosis

MI Motivational Interviewing

MTB *Mycobacterium tuberculosis*

NIAID National Institute of Allergy and Infectious Diseases

NIH National Institutes of Health

NTP South African National TB Program

PE Peer educator

PEPFAR President’s Emergency Plan for AIDS Relief

PLWH People Living with HIV

PID Patient Identifier

RA Research Assistant

sIMB situated-Information Motivation Behavioral Skills Model

SOC Standard of Care

SOP Standard Operating Procedures

TB Tuberculosis

TLD Tenofovir/lamivudine/DTG Combination Pill

UNAIDS Joint United Nations Programme on HIV/AIDS

USAID United States Agency for International Development

VAS Visual Analog Scale

WHO World Health Organization

Table of Contents

1 STUDY OVERVIEW 4

2 PERSONNEL AND TRAINING 7

3 BACKGROUND AND SIGNIFICANCE 8

4 STUDY OBJECTIVES AND AIMS 9

5 METHODS 11

6 DATA COLLECTION 15

7 DATA MONITORING AND QUALITY ASSURANCE 21

8 DATA ANALYSIS 23

9 HUMAN SUBJECTS PROTECTIONS 24

10 REFERENCES 32

1. STUDY OVERVIEW

Adaptive evaluation of mHealth and conventional adherence support interventions to optimize outcomes with new treatment regimens for drug-resistant tuberculosis and HIV in South Africa

- 1. **Summary**

The availability of shorter, more effective, entirely oral bedaquiline (BDQ) containing drug-resistant tuberculosis (DR-TB) treatment regimens (1), and highly potent, low drug-drug interaction profile, integrase strand transfer inhibitor (INSTI)-based combination ART have transformed the treatment paradigm for DR-TB HIV (2-4). However, without similar ground-breaking advances in adherence support, we will not realize the potential of these revolutionary therapeutics and novel regimens may rapidly lose their benefits due to emergent resistance to either antimycobacterial agents and/or antiretrovirals (5, 6).

- 1. **Background**

Tuberculosis (TB) is now the second leading cause of death due to a single infectious agent (7), recently displaced by SARS-Cov-2 (8). TB remains the single leading cause of death for persons living with HIV/AIDS (9). Although TB incidence is slowly decreasing (7, 10), global End TB 2020 incidence and mortality milestones were not achieved (11). An important reason for this missed opportunity is an increase in DR-TB cases with high mortality and poor treatment outcomes (12, 13). Approximately ~820,000 incident TB cases globally occur in HIV co-infected patients (7). The majority (59%) of TB patients in South Africa with known HIV status are co-infected (14, 15), and there are ~14,000 incident DR-TB HIV cases per year (7). South Africa has ~18% of global MDR-TB burden and the highest number­­­ of DR-TB HIV cases (7, 16, 17).

Medication adherence, a key predictor of outcomes in DR-TB and HIV treatment, is understudied in high burden TB-HIV settings (18-20). Patient losses during transitions in the care continuum are frequent (21), increase mortality and limit control of the linked epidemics. Demands of DR-TB HIV treatment are severe including extraordinary pill burden, severe adverse effects, lengthy treatment, isolation, and stigma with few parallels in modern medicine (22-24).

- 1. **Specific Aims**

**Aim 1. To compare the effect of a multi-arm adaptive adherence intervention on DR-TB HIV outcomes**

**Aim 1 overview**. This Aim seeks to use an adaptive implementation platform to randomize DR-TB HIV patients initiating BDQ and TLD to compare the effect of different interventions on biological and clinical endpoints (*1a*) and determine BDQ threshold adherence value associated with DR-TB culture conversion (*1b*).

**Hypothesis 1a** *In a randomized, adaptive implementation trial, the psychosocial + mHealth support arm will improve a composite DR-TB HIV clinical outcome compared to separate mHealth, psychosocial support, or enhanced standard of care arms. (N=360)*

**Hypothesis 1a** **overview.** This study aims to evaluate the impact of different adherence support elements informed by a DSD framework in patients with DR-TB HIV. Specifically, in this hypothesis we will use a four-arm, adaptive randomized design to study the impact of adherence support interventions on clinical outcome. The four arms will include: enhanced standard of care (ESOC) (I), psychosocial support (II), mHealth (III), psychosocial support + mHealth. Components of the intervention have been informed by preliminary qualitative research that elucidated critical patient needs at key stages in DR-TB HIV treatment.

**Aim 1 Hypothesis 1b** *Quantitative adherence measurement using EDM will define BDQ adherence thresholds that are more predictive for TB outcomes than conventional adherence measures. (N=180)*

**Hypothesis 1b overview** Medication adherence is widely considered to be a critical determinant of response in the treatment of infectious pathogens - including *M. tuberculosis* - but is severely understudied. For BDQ, quantitative adherence thresholds sufficient to ensure TB culture conversion at 6 months and optimal end of treatment clinical outcome are not known.

- 1. **Methods**

The overall structure is a 4-arm adaptive platform of mHealth and psychosocial adherence support interventions informed by a *differentiated service delivery* (DSD) approach. The framework is a study of mHealth and psychosocial adherence support interventions using a Bayesian adaptive design to allow comparison of elements of the intervention separately and in combination.

Participants will be randomized into one of 4 arms and followed monthly through the 6 months of intervention, then through the end of treatment telephonically, with an additional in-person visit to establish the primary outcome. Primary outcome is a combined clinical/biological outcome at 12 months described below. *Hypothesis 1a* utilizes all participants while *1b* utilizes only those in the mHealth intervention arms (3+4) since granular EDM-measured adherence is required. Detailed methods are described in section 4.3 below.

- 1. **Population**

Eligible patients will be consecutively recruited adult patients (age ≥ 18 years) presenting with all of the following inclusion criteria: (1) Culture or molecular test positive for MTB, (2) Molecular test positive for HIV or a documented HIV positive history (3) Drug-susceptibility testing by molecular (i.e. GeneXpert MTB/RIF) or conventional testing consistent with at least rifampicin-resistant TB, (4) Initiating treatment with a BDQ-containing TB regimen within 4 weeks of enrollment and first-time being treated with BDQ (5), On treatment with ART regimen, including dolutegravir-containing combination ART regimen (i.e. TLD), or starting within 4 weeks of enrollment, (5) Capacity for informed consent in either isiZulu or English.

Patients will be excluded from the study if they are prisoners, and if they are pregnant on enrollment. Patients will be recruited from King DinuZulu Hospital, a centralized TB referral hospital near Durban, South Africa which initiates the majority of BDQ treatment regimens in the province, and/or referral clinics for King DinuZulu Hospital.

- 1. **Study Duration**

Approximately two years will be allowed for enrollment. Patients will be followed until the end of treatment (approximately 4 years total).

- 1. **Participant Duration**

Study visits will occur monthly until 6 months with a follow-up visit at 12 months. A telephonic visit will occur at the end of treatment (9 to 18 months after treatment initiation DR-TB). See Schedule of Evaluations for further details.

- 1. **Potential Significance**

The availability of shorter, more effective, oral bedaquiline (BDQ) containing drug-resistant tuberculosis (DR-TB) treatment regimens (1), and highly potent, low drug-drug interaction profile, integrase strand transfer inhibitor-based combination ART have transformed the treatment paradigm for DR-TB HIV (2-4). However, without similar ground-breaking advances in adherence support, we will not realize the potential of these revolutionary therapeutics and novel regimens may rapidly lose their benefits due to emergent resistance to either antimycobacterial agents and/or antiretrovirals (5, 6).

The NIAID has identified improved treatment of DR-TB and TB/HIV as a research priority (25). The proposed research will increase *local scientific capacity* in South Africa and train local investigators, including women and minorities (26). Successful completion of our Aims will substantially advance our understanding of DR-TB and HIV adherence dynamics in an integrated research strategy land program and develop an innovative set of tools for the assessment and support of dual TB and ART medication adherence. *Conceptually*, our work will advance the *differentiated service delivery* model into DR-TB HIV treatment. *Practically*, we will evaluate components of adherence support using an adaptive platform to determine the effectiveness of mHealth and psychosocial support (*Aim 1*). We will characterize potential intervention mechanisms of action by measuring model-based socio-behavioural variables and contextualize the longitudinal intervention impact in key stages of DR-TB HIV treatment using qualitative methods.

The study findings may be used to inform programmatic management and the development of further interventions that promote adherence for DR-TB HIV patients.

1. PERSONNEL AND TRAINING
   1. **Roles**

Roles of Lead Investigators: Investigators will provide leadership and mentorship, supervise the study team and monitor the project, develop the study protocol. Drs. Naidoo and O’Donnell are the co-Principal Investigators of this study.

Dr. O’Donnell with Dr. Naidoo will be responsible for the overall conduct of the study and will supervise all study related activity.

- 1. **Staff Training**

All study staff will have current good clinical practice (GCP) training. The study timeline will include 6 months for training study staff. Training in study protocols will be performed prior to recruitment of patients and refresher training will be performed regularly.

Dr. Cheung will lead a training on the randomization strategy and software use. Study staff assigned to randomization duties will participate in training prior to study initiation.

Supervision will be provided to ensure participant safety and good study conduct.

**Enhanced standard of care (Arm 1)** will include usual care as administered by hospital and clinic staff enhanced by study staff-provided treatment literacy and study-provided training for treating physicians, nurses, pharmacists, and social workers prior to study initiation and periodically with refresher trainings

1. BACKGROUND AND SIGNIFICANCE

Tuberculosis (TB) is the leading cause of mortality for people living with HIV, causing over 200,000 deaths annually (7). Approximately 860,000 incident TB cases occur in HIV co-infected patients worldwide (27). In southern Africa, interaction between TB and HIV epidemics has led to increased community transmission of drug-resistant TB (DR-TB) (17, 28-30),critically undermining TB and HIV-related treatment goals (31, 32).

In South Africa, there are approximately 14,000 incident DR-TB HIV cases per year (7). Antiretroviral therapy (ART) in DR-TB HIV is challenged by older regimens and drug-drug interactions. Dolutegravir, an integrase strand transfer inhibitor, formulated as once-daily combination ART (tenofovir/lamivudine/dolutegravir (TLD)) (33, 34), is recently available in South Africa (35). TLD is superior to older comparator regimens, protective for ART discontinuation, and proposed as first-line ART including for treatment of DR-TB HIV (36, 37), but adherence in this context is not well studied.

**Medication adherence** is a key predictor of TB treatment outcomes and emergent drug-resistance (38-40), and is severely understudied in high burden TB/HIV settings (41, 42). DR-TB HIV treatment demands are daunting, including high pill burden, adverse effects, lengthy treatment, and stigma (23, 43, 44) with challenges in key treatment stages (45, 46). Bedaquiline (BDQ), a highly effective antimycobacterial, is the first new DR-TB drug in 40 years, and WHO-recommended as a key component of all new DR-TB treatment regimens (1, 47). Research by our team in South Africa, shows high mean BDQ adherence in DR-TB HIV measured using cellular-enabled EDM (48). However, a substantial adherence challenged subgroup (~16% patients) is at high risk for treatment failure, mortality, and emergent bedaquiline resistance (1, 49).

Patient-centered adherence support strategies using psychosocial support and mHealth (health practices supported by mobile technologies and devices) may improve DR-TB HIV outcomes. These modalities have improved medication adherence in HIV (50-55), hypertension (56, 57), diabetes (58), and psychiatric disease (59-61). Our team has done important early work in mHealth adherence support and comprehensive psychosocial support for DR-TB HIV (48, 62). A critical gap, addressed by our proposal, is to understand the relative contributions of mHealth and psychosocial adherence support to improve clinical and biological outcomes in DR-TB HIV treatment.

**Differentiated service delivery (DSD)** is an innovative person-centered care model tailored to the health status and clinical needs of persons living with HIV/AIDS, informed by social, behavioral and structural factors (63-65). DSD methods include *mHealth* (health practices supported by mobile technologies), psychosocial support, and community-based care (65, 66). We have piloted mHealth-guided adherence support in DR-TB HIV treatment in South Africa, using electronic dose monitoring (EDM) to measure real-time adherence (48).

For our study in, we use an adaptive design including patient-centered care concepts derived from DSD. Using elements of the DSD approach within each of the 3 intervention arms (excluding the enhanced standard of care arm) the intensity of each intervention will be calibrated based on empirically determined participant requirements. DSD evaluation will be performed by incorporating monthly questions regarding DSD acceptability and patient preference, intensity of DSD inputs will be recorded, but due to limited resources a statistical power stratified analysis by DSD will not be performed.

**The burden of stigma for patients with DR-TB/HIV** Stigma was initially described by Goffman as a “deeply discrediting” attribute understood through a “language of relationships” and social interactions (67). Contemporary scholars have called attention to social interactions, structures and practices that set up normative expectations about what is acceptable versus devalued (68, 69). Stigma characterizes the lived experience of HIV and is acknowledged as a key obstacle to global HIV control (69). Health activists and persons with HIV have confronted HIV stigma (70) to protect patients from systemic discrimination (71). Persons with TB have long been stigmatized due to their association with poverty and the fear of contagion (72, 73). High rates of HIV co-infection in countries such as South Africa and emergence of nearly untreatable DR-TB, have renewed fears about TB patients leading to new forms of TB-HIV stigma (71, 74, 75). Intersectional DR-TB/HIV stigma has been identified by our group as an important obstacle to treatment success (76).

1. STUDY OBJECTIVES AND AIM
   1. **Rationale**

Improved treatment of drug-resistant tuberculosis and HIV has been identified as a research priority (25). Implementation of the project will increase local scientific capacity to conduct implementation research by 1) developing programmatic links between central hospitals and de-centralized, community-based treatment programs for drug-resistant TB-HIV, 2) building on existing President’s Emergency Plan for AIDS Relief (PEPFAR) program strengths using the Centre for the AIDS Programme of Research in South Africa’s (CAPRISA) excellent research infrastructure to generate evidence-based recommendations, 3) training and developing junior researchers in implementation science and mixed methods approaches (26).

We will implement an adaptive trial for improving adherence and retention. We will explore the impact of the intervention on reducing barriers and improving facilitators to adherence and qualitatively explore the feasibility and acceptability of an integrated intervention for adherence and retention in care. We will carry out this work in the inpatient setting by expanding the standard of care and focusing on the transition to outpatient care. We will also extend the intervention to focus on the outpatient community setting where patients may have the greatest adherence challenges and highest likelihood of treatment default.

- 1. **Specific Aim and Hypotheses**

**Aim 1 overview**. This Aim seeks to use an adaptive implementation platform to randomize DR-TB HIV patients initiating BDQ and TLD to compare the effect of different interventions on biological and clinical endpoints (*1a*) and determine BDQ threshold adherence value associated with DR-TB culture conversion (*1b*).

**Aim 1. To compare the effect of a multi-arm adaptive adherence intervention on DR-TB HIV outcomes** Using a 4-arm Bayesian, adaptive trial design (77), we will prospectively evaluate adherence support components in DR-TB HIV patients treated with BDQ and TLD. The primary outcome will be a composite of HIV viral load, TB culture conversion, survival, and retention in care at 12 months. Within each intervention arm, required adherence support will be empirically determined and delivered using a DSD framework.

**Hypothesis 1a** *In a randomized, adaptive implementation trial, the psychosocial + mHealth support arm will improve a composite DR-TB HIV clinical outcome compared to separate mHealth, psychosocial support, or enhanced standard of care arms. (N=360)*

**Aim 1 Hypothesis 1b** *Quantitative adherence measurement using EDM will define BDQ adherence thresholds that are more predictive for TB outcomes than conventional adherence measures. (N=180)*

5 METHODS

- 1. **Overview**

Time line

*Dates are approximate*

- 6 months for staff training
- Obtain regulatory and ethical approvals
- Approximately 36 months for enrolment (360 patients)
- Participants will be followed monthly until 6 months
- Follow-up visit at 12 months
- End of treatment telephonic visit

Overview of Study Processes (see 6.1 Schedule of Study Evaluations)

*All participants*

- Informed consent
- Baseline (Intake) Assessment
- Social and Medical history, etc.
- Monthly and Follow-up Assessments
- Sputum and blood collection
- Collect HIV viral loads, sputum cultures through routine clinical care
- Collect monthly pharmacy records
- Determine clinical end of treatment outcome

*Arm 1 Enhanced Standard of Care*

- Usual clinical care enhanced by:
  - Treatment literacy counselling
  - Monthly physician visits
  - Social work and other services initiated routinely
  - Study team provides extra training for physicians, nurses and social workers to enhance standard of care

*Arm 2 Psychosocial Support*

In addition to Arm 1:

- Discharge planning (if inpatient)
- Community treatment planning (if outpatient)
- Individual counselling
- Community adherence support group
- Home visits*
- Monthly TB adherence assessment questionnaire Wilson’s adherence scale**

*Arm 3*^$^ *mHealth*

In addition to Arm 1:

- Weekly text messaging*
- Wisepill RT3000 devices: one for bedaquiline and one for ART
- Troubleshooting phone calls for detected non-adherence*
- Weekly assessment of adherence measured by the Wisepill RT3000 device^$^

*Arm 4 ***^$^*Psychosocial support + mHealth*

Combination of Arms 2 and 3^$^

- Discharge planning (if inpatient)
- Community treatment planning (if outpatient)
- Individual counselling
- Community adherence support group
- Home visits*
- Monthly TB adherence assessment questionnaire Wilson’s adherence scale**
- Weekly text messaging*
- Wisepill RT3000 devices: one for bedaquiline and one for TLD
- Troubleshooting phone calls for detected non-adherence*
- Weekly assessment of adherence measured by the Wisepill RT3000 device^$^

*These elements may be increased in intensity or frequency depending on empirically assessed patient needs within each arm

**If the participant has Wilson’s adherence scale **≥** 3 he/she will be considered ‘at-risk’ for non-adherence and will have the intensity of the intervention increased in the following stepwise fashion: A) Telephonic check in by study staff B) Increased frequency of counselling sessions from monthly to biweekly C) home visit by a multidisciplinary study team.

^$^Less than 90% observed/expected doses will be considered at risk for non-adherence and will have the intensity of the intervention increased in a stepwise fashion: A) Telephonic check in by study staff B) Increased frequency of text messaging from weekly to daily.

*Optional substudy: In-depth Interviews*

Approximately 12 participants from each arm will be seleted for an in-depth interview. Participants will have the option to opt-out at the time of consent. Purposive recruitment (155, 156) based on age and gender will explore diverse viewpoints and pathways for intervention impact. Two IDIs will be conducted with each participant (157, 158): the first in Month 2-3 of DR-TB treatment to capture pathways of intervention impact during the early stages of treatment (initiation and intensive treatment phase), and a subsequent IDI in Months 6-9 to evaluate any pathways during later stages (continuation and late treatment phase). IDIs will be conducted in the participant’s native language, following semi-structured guides. The session will be recorded.

- 1. **Study Design**

This study will follow a 4-arm Bayesian, adaptive trial design. As patients are enrolled, they will be randomized into one of the four arms.

The study will be carried out within a common structure to allow for efficient enrollment and analysis. The overall structure is a 4-arm adaptive platform of mHealth and psychosocial adherence support interventions informed by a differentiated service delivery (DSD) approach.

Aim 1 is an adaptive study of mHealth and psychosocial adherence support interventions using a Bayesian adaptive design to allow comparison of elements of the intervention separately and in combination. Aim 1 participants will be randomized into one of 4 arms and followed monthly through the 6 months of intervention, then through the end of treatment telephonically, with an additional in-person visit to establish the primary outcome. Primary outcome is a combined clinical/biological outcome at 12 months described below. Hypothesis 1a utilizes all participants while 1b utilizes only those in the mHealth intervention arms (3+4) since granular EDM-measured adherence is required.

- 1. **Population and Study Setting**

These studies will be conducted within the established implementation science research infrastructure at the CAPRISA Treatment Clinical Research Site in Durban, South Africa which has a track-record of performing high quality, impactful implementation science studies (78, 79).

- 1. **Recruitment**

This is a prospective interventional cohort study for people with HIV that have been diagnosed with DR-TB and are initiating treatment at King DinuZulu Hospital (KDH), a centralized TB referral hospital in Durban, South Africa, which initiates the majority of BDQ treatment regimens in the province, and other affiliated clinical sites. Eligible patients include those from referral clinics for King Dinuzulu Hospital. Consecutive patients meeting inclusion criteria will be approached for enrolment into the study by a member of the study staff. Patients willing to participate will complete an Informed Consent form.

- - 1. Inclusion Criteria

1. Age ≥ 18 years
2. MTB culture positive with at least rifampicin resistance **OR**

Molecular drug susceptibility test confirming resistance to at least rifampicin resistance **OR** Polymerase chain reaction test (Xpert MTB/RIF) result showing MTB positive and RIF resistance.

1. Initiating treatment for DR-TB which includes Bedaquiline (BDQ) containing regimen within 4 weeks of enrollment and first-time being treated with BDQ.
2. Have capacity for informed consent.
3. Participant has a known HIV infection.
4. On ART regimen, or plans to initiate ART within 4 weeks of enrollment, as per clinician recommendation.
   - 1. Exclusion Criteria

Pregnancy

Prisoners

Discretion of IOR or clinician

- 1. **Randomization Strategy**

Eligible participants will be assigned to one of four intervention arms at baseline using a two-step randomization. In the first step, participants will be randomized to (Arm 1) vs (Arms 2-4) in a 1:3 ratio, using a minimization method to achieve balance over the following baseline stratifying variables: inpatient vs. outpatient treatment; MDR-TB vs. higher level MTB drug resistance; baseline CD4 T-cell count >200. In a second step, a Bayesian adaptive randomization (BAR) scheme will be used to further randomize the DSD participants to one of three DSD arms: psychosocial support (Arm 2), mHealth support (Arm 3), or mHealth + psychosocial support (Arm 4) based on the posterior distribution of the success rates of the three arms. Specifically, the randomization probability to an arm will be proportional to posterior probability that arm has the highest success rate (80, 81). For example, the randomization probability to Arm 2 (psychosocial support alone) will be calculated as ***Prob*** *(****P_2_ > P_3_ and P_2_ > P_4_ | data****),* where *P_2_, P_3_, P_4_* denote the success rates of achieving the combined treatment outcome; in Arms 2, 3, 4 respectively, and data are updated continuously throughout the study. Under this randomization scheme, **90 participants** will receive ESOC and **270 participants** will receive random allocation among the three arms (**360 participants total**). In addition, we will adopt some practical implementations of the BAR scheme. First, we will apply an initial run-in period in which DSD participants will be assigned with equal probability to the three arms. The run-in period will end once the first 30 DSD participants have completed their 12-month evaluation. Second, while the primary endpoint is 12-month outcome, we will incorporate outcome at 6-months in updating the randomization probability during the study (81, 82). Third, web applications will be used to facilitate adaptive randomization in real time with up-to-date data. Intermediate outcomes at 6-month, the final 12-month combined treatment outcomes, and the stratifying factors will be updated on a continuous basis by the study coordinator via a data entry app. Randomization probabilities will be updated based on the updated data; and the study coordinator will obtain randomization code based on these updated probabilities via a randomization app.

1. DATA COLLECTION
   1. **Schedule of Study Evaluations**

| **Procedures^+^** | **Baseline (enrolment)** | **Month 1** | | **Month 2** | | | | **Month 3** | **Month 4** | | | **Month 5** | **Month 6** | | | | **Month 12** | | **End of Treatment**  **(6-18 months)** |
| --- | --- | --- | --- | --- | --- | --- | --- | --- | --- | --- | --- | --- | --- | --- | --- | --- | --- | --- | --- |
| **All Participants** |  | | | | | | | | | | | | | | | | | | |
| Informed consent | x |  | |  | | | |  |  | | |  |  | | | |  | |  |
| Randomization to group (Arms 1-4) | x |  | |  | | | |  |  | | |  |  | | | |  | |  |
| Treatment literacy counselling | x |  | |  | | | |  |  | | |  |  | | | |  | |  |
| Baseline (Intake) Assessments | x |  | |  | | | |  |  | | |  |  | | | |  | |  |
| Complete medical history chart abstraction | x | x | | x | | | | x | x | | | x | x | | | | x | | x |
| Follow-up Assessments |  | x | | x | | | | x | x | | | x | x | | | | x | | x |
| Early morning sputum sample collection for storage | x | x | | x | | | |  |  | | |  | x | | | | x | | x |
| Blood Sample collection for storage | x |  | | x | | | |  |  | | |  | x | | | |  | |  |
| Depression Screen | x |  | |  | | | |  |  | | |  | x | | | | x | |  |
| Stigma Screen | x |  | |  | | | |  |  | | |  | x | | | | x | |  |
| sIMB Questionnaire | x |  | |  | | | | x |  | | |  | x | | | | x | |  |
| Post Trial call |  |  | |  | | | |  |  | | |  |  | | | |  | | X |
| **Intervention groups only** |  | | | | | | | | | | | | | | | | | | |
| **Arm 2** |  | | | | | | | | | | | | | | | | | | |
| TB adherence assessment (WAS) |  | x | | x | | | | x | x | | | x | x | | | | x* | |  |
| Monthly counseling intervention (MCI)^$^ |  | x | | x | | | | x | x | | | x | x | | | |  | |  |
| Adherence Support Groups (ASG) |  | x | | x | | | | x | x | | | x | x | | | |  | |  |
| Home visits^&^ | As needed basis | | | | | | | | | | | | | | | | | |  |
| Post hospital discharge phone call (PDP) |  |  | |  | | Within 7 days post hospital discharge | | | | | | | | |  | | |  |  |
| **Arm 3** |  | | | | | | | | | | | | | | | | | | |
| Pill count by Wisepill electronic pillbox | Daily from time of randomization into intervention arm until discharge from study. | | | | | | | | | | | | | | |  | | | |
| TB adherence assessment (Wisepill) |  | | x | | x | | x | | | x | x | | | x | | | | x* |  |
| Issue Wisepill device, training | Within 14 days post-randomization into the intervention arm. | |  | |  | |  | | |  |  | | |  | | | |  |  |
| Adapted Wisepill Intervention (AWI) | Intervention (phone call) triggered by non-adherence as detected by Wisepill non-opening. | | | | | | | | | | | | | | | | | | |
| **Arm 4** |  | | | | | | | | | | | | | | | | | | |
|  | Will include all available interventional tools** | | | | | | | | | | | | | | | | | | |
| **Optional Sub-study**  **In-Depth Interviews (IDI)^#^** |  | | X | | | |  | | | | X | | | |  | | | | |

^+^Monthly pharmacy records will be obtained; Routine HIV viral loads, sputum culture results will be collected from the clinic

*If participant remains on treatment

^$^Frequency may increase based on need

^&^Participant may opt out. Timing and frequency based on the discretion of the investigator

**Arm 4 is the combination of each intervention. Participants that are randomized to Arm 4 will complete all tasks at each time point specified for Arms 1-3.

^#^A sub-set of participants from each arm will be selected to participate in an IDI. Participant may opt-out. Two interviews should occur, one between months 2-3 and the second between months 6-9.

- 1. **Study Data and Sample Collection**

*All participants*

All participants will receive *Enhanced Standard of Care (*ESOC).

Enhanced Standard of Care:

ESOC will consist of care from trained and supported physicians, nurses, and social workers who have received repeated trainings from study staff on medical and behavioral aspects of DR-TB HIV care, which will be documented in terms of date, attendance, and content.

All participants who receive care as inpatients will receive an orientation to DR-TB treatment in form of a group session designed to impart key behavioral information and health knowledge about the disease, treatment, and skills to obtain optimal outcome.

All participants will receive pamphlets and informational materials designed to support the above goals.

All participants who receive care as inpatients will receive an exit counseling session scheduled prior to discharge where the study staff will review key behavioral information and health knowledge about the disease, treatment, and skills as well as review challenges to reproducing care as an outpatient.

Study participants will complete study assessments at baseline (enrollment) and monthly for the first six months. Ideally follow-up questionnaires will be completed in person but allowance will be made for telephonic completion of questionnaires. Additional questionnaires will be completed following discharge (community treatment follow-up) and at the end of treatment. See section 6.1 for additional details.

Psychosocial support methods

Psychosocial support methods include individual counseling/case management, home visits (if warranted and consented to), and adherence support groups which can support patient motivation and provide information to assist behavioral change. Participants in arms 2 and 4 will participate in individual counseling aligned with their monthly clinic visit, or more frequently if indicated. Individual counseling will use motivational interviewing (MI) techniques, based on the 4-part engaging, focusing, evoking and planning approach for each participant to set goals and anticipate barriers to successful engagement in care (83). Counselors will be qualified mental health counselors or credentialed social workers trained in MI skills using validated training materials (84). If required, the same trained counselors known to patients would conduct home visits. To minimize potential stigma and increase confidentiality, home visitors would arrive in unmarked vehicles and discuss the visit only with the patient or agreed upon contacts. Participants in arms 2 and 4 will attend monthly, gender specific, structured adherence support groups. Adherence support groups will be facilitated by counselors trained in group facilitation methods; group curriculum will include 6 sessions that focus on practical topics including accessing social grants, economic stability, and family issues and will utilize guest speakers as appropriate. Structured support groups have been used in patient-centered healthcare to address both support and information needs among similarly affected individuals (85). Gender specific groups have been found to promote open sharing and allow for focus on gender specific topics (86).

mHealth Methods

The portable Wisepill RT3000 cellular-enabled electronic pill boxes (‘Wisepill’) uses 2G/3G cellular network to automatically synchronize with the Wisepill Cloud service and was designed for use in research and clinical trials. Participants will receive 2 Wisepill devices and training on loading, charging and storage. One Wisepill will be designated and externally labeled for ART and the other device for BDQ, to avoid confusion. Pill box openings serve as a surrogate for adherence to ART (which may be either TLD, or NVP or LPV/RTV) and BDQ respectively. Each participant will select a text message reminder from a guided menu of choices and receive a weekly text message encouraging adherence. For bedaquiline **≥** 1 missed openings or openings outside of the programmed dose-window (not due to technical issues) over a 2 weeks period for the participant will receive a semi-scripted study call to support regular adherence. For Bedaquiline **≥** 1 will increase the intervention as described below. For ART **≥** 2 missed openings over 2 weeks period the participant will receive a semi-scripted study call to support regular adherence. For ART **≥** 2 missed openings will increase the intervention.

For ART **≥** 2 missed opening over 2 weeks period the participant will receive a semi-scripted study call to support regular adherence. For ART **≥** 2 missed openings will increase the intervention.

*Reimbursement*

Patients will be reimbursed for study visits at baseline, monthly 1-6 months, 12 months, and end of treatment. If the patient participates in the optional IDI, he/she will be reimbursed for each IDI session that he/she completes. Participants will be reimbursed for the Adherence support groups visits.

**Clinical Data Elements**

Data for the study will be collected at the following visits: a) baseline (enrolment) visits, b) monthly clinical visits, c) follow-up or end of treatment visits, and d) at community adherence support groups. See Appendix for all questionnaires. Questionnaires will be administered by study staff fluent in both English and isiZulu.

A standardized data collection instrument will be used in this study.

*Clinical Study visits*

Baseline (enrolment) visits: Data will be abstracted from patient charts and medical history. Study staff will administer baseline (intake) assessment collecting sociodemographic characteristics, medical history, knowledge, attitudes and beliefs. These data will include date of TB treatment initiation, date of ART initiation, information regarding past history of TB, smear and culture results.

Participant Contact Information: At the time of study enrolment, participants will disclose their names, addresses and phone numbers so that they may be contacted by the study staff regarding study visits. Sensitive data will be kept separately from study materials and will not contain patient study identifiers. This will be updated at monthly visits.

Telephonic study visits

Follow up visit (month 1,3,4,5, end of treatment) will be conducted telephonically in the event the participants are unable to attend the study visits.

Electronic Data: Participants will be provided with electronic pillboxes at baseline (enrolment). Electronic data regarding adherence will be collected monthly until Month 6.

End-of-treatment Interview: When participants complete treatment, he or she will complete an interviewer-administered standardized questionnaire to collect data including knowledge, attitudes, adherence, treatment acceptability, beliefs and outcome.

In-depth Interview Sub-study: Two IDIs will be conducted with each participant (87, 88): the first in Month 2-3 of DR-TB treatment to capture pathways of intervention impact during the early stages of treatment (*initiation* and *intensive* treatment phase), and a subsequent IDI in Months 6-9 to evaluate any pathways during later stages (*continuation* and *late treatment* phase). IDIs will be conducted in isiZulu/English by a trained qualitative interviewer not involved in other aspects of the study.

- 1. **Biological Specimen Collection, Preparation, Handling and Shipping**
     1. **Biological Specimen collection**

In addition to a medical chart review, participants will have specimens collected.

At the baseline (enrolment) visit, specimen collection may include:

- 22.5 ml blood

- Two sputum samples

At month 2 & 6 additional specimen collection may include:

- 14 ml blood

- One sputum sample

At month 1 ,12 & at the end of treatment

-One sputum sample.

The biological specimens will be stored for future studies of transcriptomic and immune biomarkers of TB treatment response.

The specimens will be coded with the patient identifying number and stored separately from any personal or sensitive information or link to the consent form. The investigators will receive only the coded specimens.

Participants will be followed-up for additional specimen collection:

See Section 6.1.

- - 1. **Laboratory Evaluations**

*Blood*

-HIV viral load, biobanking for future studies including metabolomics, proteomics, biomarkers, and transcriptomics

*Sputum*

Sputum tests may include but are not limited to:

- Culture smear microscopy

- Rapid test for isoniazid, rifampicin and fluoroquinolone resistance

- First and second line drug resistance testing

- Rapid molecular diagnostics (eg. Hain and GeneXpert)

- Nucleic acid amplification testing

- Pathogen whole genome sequencing

1. DATA MONITORING AND QUALITY ASSURANCE
   1. **Statistical and Data Management**

Prior to enrollment, all research staff will participate in human subjects protection training/Good Clinical Practice training to ensure sensitive data confidentiality for all study participants. Informed Consent Forms and all forms containing patient identifiers will be kept separate from study forms in a secure, locked location. Upon enrolment, participants will be assigned a unique study identifier (PID) assigned by the CAPRISA Data Management Center. Relevant clinical data will be collected by chart abstraction. Baseline (enrolment), Monthly Clinical Visits, and Follow-up Interviews will be collected with tablet-based collection forms (Case Report Forms (CRFs)). The PID will be used on all CRFs to identify the participant for the duration of the study.

Prior to capturing any study data into CRFs, instructions will be given by the CAPRISA Data Management core. Completed CRFs must be checked by the Quality Control (QC) officers. CAPRISA Data Managers will verify and validate patient data. Quality control reports are produced and approved per CAPRISA data management Standard Operating Procedures (SOPs).

RedCap software will be utilized for the development of study forms, data entry, and data management of electronic data. Electronic data will be kept securely on encrypted and password protected end point devices with support from the CAPRISA Data Management Core. Users on the study team will have access to the study database with individual login credentials including username and password.

Only the designed members of the study staff will have access to the key linking the study PID data to patient identifiers.

- - 1. **CAPRISA Data Management Core**

The Data Management Core comprises 3 components: a) IT section, b) DataFaxing, data entry and data encoding and c) data management. Skills, technical support, and infrastructure to enable quality data collection and efficient transfer of data from the sites to the data management center are available through the CAPRISA data management core. They are also responsible for purchase and maintenance of all data management equipment such as the DataFax machines.

- - 1. **Quality Control/Assurance**

Quality checks will be performed on the data entered into the RedCap database.

-Responses are clearly documented within designated spaces.

-All fields are completed with participant data; if no data was available, this is specified.

-The participant PID is recorded on all pages of the study forms.

-The CAPRISA laboratory manager will ensure that all involved laboratories are compliant with Good Laboratory Practices (GLP).

- The CAPRISA pharmacist will provide oversight for the preparation of the electronic pillboxes and pill counts.

QA/QC of data will be undertaken according to CAPRISA SOPs.

- - 1. **Electronic Data Storage**

CAPRISA It is the responsibility of the CAPRISA data management core to assure the quality of computerized data for the study. Study staff will be trained in source documentation requirements in accordance with the study SOP for Source Documentation and in proper forms completion techniques.

Database files will be password-protected, and access to the files will be limited to authorized study staff members only. Quality control and validation checks will be performed and data cleaning steps will be undertaken prior to finalizing the study database for analysis. Analyses will be conducted by study statisticians.

- - - 1. **Long Term Data Storage**

Electronic copies of study CRFs and related documents will be stored securely both during and after study completion. During the study, the original completed forms for each participant will be kept on-site at the CAPRISA KDH site in accordance with federal (both NIH and US guidelines). Upon completion of the study, and finalization of the database for analysis, any original, paper-based forms will be bound and kept off-site (separate site) for long-term storage. Personal information collected in this study will be archived in accordance with applicable guidelines and laws, including the Protection of Personal Information Act, 2013. CAPRISA has a standing agreement with a document storage company to archive large amounts of documents. CRF data on the RedCap server will be accessible to the study staff and the statistician in a read-only mode. The data management team will have write-access, with access being restricted by passwords and validation levels. Study staff that has access to the data on the computer systems will be trained in how to access the system and the importance of system security. All information will be backed-up at regular intervals, and backups will be stored in file cabinets or secure areas with limited access

Columbia University Medical Center Data transported to Columbia University will be sent as coded with the PID. Only the South African study designees will have access to the key. Study personnel at Columbia University will not have access to identifying or sensitive patient data at any point. Study data will be kept in electronic format and will be kept confidential in accordance with Columbia University Medical Center guidelines <http://www.columbia.edu/acis/security/users/index.html>.

- 1. **Instructions for Biological Specimen Management**

Maximal infection control precautions will be taken by the research team members during specimen collection and preparation including use of double gloves, safe venipuncture equipment and a fit-tested N-95 respirator during sputum induction. Specimens will be collected in designated containers and labeled with a printed, barcoded labels that contain the patient identification number, the study visit, the specimen type, the intended assays and the specimen destination. Specimens for testing at the commercial laboratories will be directly transported there by courier.

Specimens for processing at the CAPRISA laboratory will be placed on ice and transported by courier to the CAPRISA laboratory where they will be appropriately aliquoted.

- - 1. **Biohazard Containment and Specimen Shipment**

All specimens will be labeled by barcode and identifiable by PID. A specimen tracking log (including attestation of appropriate temperature maintenance during transport) will be utilized to document all transport of specimens between sites.

Transmission of HIV and other pathogens can occur through contact with contaminated needles, blood, blood products, and other secretions; appropriate blood and secretion precautions will be employed by all personnel in the drawing of blood and collection of other specimens and shipping and handling of all specimens for this study, in accordance with guidelines by the Centers for Disease Control and Prevention and the National Institutes of Health.

All dangerous materials, including diagnostic specimens and infectious substances, must be transported using packaging mandated by CFR 42 Part 72. Please refer to instructions detailed in the International Air Transport Association (IATA) Dangerous Goods Regulations.

- - 1. **Future Use of Stored Specimens**

A portion of the blood or sputum may be frozen and kept at CAPRISA for future research. A portion of these specimens may be kept for 10 years for assays developed in the future in accordance with NIH polies and practices.

Specimens will be stored confidentially with the barcoded patient identifier, separately from the key or any other identifying or sensitive patient information. The Biomedical Research Ethics Committee will monitor and provide permission for their ethical use in the future.

Specimens may be shipped at a later date. The appropriate regulatory documents will be completed and submitted prior to shipment.

1. DATA ANALYSIS
   1. **Power Calculations and Statistical Methods**

Hypothesis 1a

The study is expected to enroll a total of 360 participants, who will be randomly assigned to one of four intervention arms. Since retention in care is a component of the composite 12-month outcome, we expect that missing data will be minimal. Since ‘missingness’ will be part of the primary outcome (i.e., lack of retention in care or loss to follow up) patients who are ‘lost’ will actually provide important study data. A Bayesian adaptive randomization algorithm will be used to assign interventions to patients to improve the power of detecting any superior arm(s) and to increase the number of patients treated in the better arm(s) during the study.

Hypothesis 1a

Assuming EDM have an accuracy of 85% (for either positive and negative outcome) against a random guess (i.e., a null 50% accuracy), the statistical power for a one-sample binomial test (two-sided at 5% level) will be greater than 99% when the sample size available for this aim is 90; while we expect the sample size available for this aim will be random due to BAR and will be at least 180.

1. HUMAN SUBJECTS PROTECTIONS
   1. **Ethical Considerations**

This protocol and supporting documents will be submitted concurrently to the HRPO IRB at Columbia University and BREC ethics boards.

The study will be conducted in compliance with South African, US, national and local regulations and guidelines applicable to research involving human subjects, and in accordance with the International Conference on Harmonization (ICH)/Good Clinical Practice (GCP). Should regulations and guidelines differ between countries, the more restrictive regulations and guidelines will apply. The protocol, informed consent forms, and study materials to be completed by study participants will be reviewed by the ethical review boards at Columbia University and CAPRISA. Modifications to any study materials will be submitted to regulative authorities as necessary and on an ongoing basis.

- 1. **Human subjects considerations**

The investigators are committed to the protection of the rights of all participants in the proposed research, in accordance with USAID policy. Prior to study enrolment, all research staff will be trained in principles of human subjects protection and management of confidential study materials. Study consent forms and protocols, including intervention materials, questionnaires, and data abstraction tools will be approved by the Human Research Protection Office Institutional Review Board (IRB) at Columbia University Medical Center and BREC.

- - 1. Exclusion of Subpopulations. Children under the age of 18 will be excluded. It is likely that factors associated with ART and TB medication initiation and retention in children will vary from that of adults and subsequently would obscure the sensitivity of the analysis.
    2. Vulnerable Populations. Pregnancy is an exclusion criterion for this study as risks associated with drug resistant TB treatment are different for pregnant women. Prisoners will be excluded from this study.
  1. **Informed Consent Process**

Only participants providing informed consent per IRB/IEC requirements will be enrolled in this study. Informed Consent Process will begin with a concise and focused presentation of the key information about the research study. A member of the study staff will explain the protocol and informed consent documents prior to obtaining informed consent in the preferred language of the potential participant (English or isiZulu). The informed consent document contains information on the study purpose, study procedures, possible risks/discomforts, possible benefits, alternatives, confidentiality, compensation for participation, and rights (including the right to withdraw from the study) and will provide contact information to the study staff and human protection offices. Potential participants will have the opportunity to discuss the protocol and address questions with a member of the study staff. Participation in the study is voluntary and refusal to participate will not affect the quality of care that individuals receive. Participants will be informed that he or she may withdraw from the study at any time. Withdrawal will not affect the quality of care that he or she receives.

When the potential participant fully understands the details of the study, his or her rights and responsibilities and expresses a wish to participate, the participant and the person explaining consent will sign and date the informed consent form. The person will be provided with a copy of the consent form as a record. If the participant chooses to withdraw at any time, he or she may simply contact the person obtaining consent or another member of the study team.

There will be a separate section detailing risks and benefits of permitting biobanking of serum/plasma for future studies including metabolomics, proteomics, and transcriptomics.

Participants will be given the opportunity to decline participation in the biobanking component and still participate in the overall study.

- 1. **Confidentiality**

Every effort will be made to ensure that participant information will remain confidential and, to the extent permitted by applicable laws and/or regulations will not be made publicly available. To prevent breaches of confidentiality, participants’ identifying data will be coded to a patient identifying number. Personal and identifying information will be kept separately and securely from study forms and study databases. Only the South African PI and the study team at CAPRISA will have access to the data key and identifier-containing documents. In accordance with the law, data may be reviewed by representatives of the IRB/IEC and individuals tasked with duties of monitoring and quality assurance.

Paper forms used to collect study data will be stored in secure locked cabinets. Electronic forms used to collect study data will be encrypted and accessible only through a password protected portal. Data from questionnaires and study materials will be entered into electronic databases.

Both paper study forms and electronic database data will be identifiable only by participant ID. Electronic databases will be stored on encrypted and password protected endpoint devices in accordance with guidelines. Study staff will be the only persons with access to the study database and paper records.

- 1. **Quality Assurance**

The study will be conducted in compliance with the protocol, Standard Operating Procedures (SOPs), Good Clinical Practice (GCP), and applicable regulatory requirements in South Africa. All relevant study documents, including recruiting materials, informed consent documents and the protocol will be approved by the local ethics board. CAPRISA’s Quality Assurance team will ensure compliance with applicable regulations and ethical standards regarding protocol compliance, proper completion of informed consent procedures, eligibility verification, source documentation collection and maintenance, and CRF completion.

The study coordinators based in South Africa and US will hold bi-weekly teleconferences with members of the Quality Assurance team to review data and procedures. Additional external monitoring will occur quarterly by Dr. O’Donnell and the US-based Study Coordinator. External monitoring will include review of all study documentation, collected data and study team performances. QC reports will be available from the CAPRISA Data Management Core, which will be reviewed periodically.

- 1. **Potential Risks and Protections**

This study is of minimal risk. There is no drug or device intervention involved with this study. This study involves a behavioral intervention and increased monitoring of adherence to both TB medications and ART. Participants will answer questions about the standard care they receive. Below are possible risks associated with participating in the study and protections against them.

Loss of confidentiality: There is a risk of loss of confidentiality throughout the course of the study. Every effort will be made to keep study participants’ identifying information confidential. Personal identifiers (including name, address) will be kept in a separate and secure location from data collected for the purpose of the study. Participants will be provided with a study ID; all forms will use the PID for identification. Only the South African study team will have access to the key to decode the study ID.

All study forms will be identified with only the study ID. Study documents will be accessible only by study personnel. Paper study forms will be kept securely in locked cabinets; electronic study forms will be secured on an encrypted and password protected study portal. Study databases will be kept on encrypted and password protected end point devices in accordance with privacy guidelines.

Staff will be trained on Good Clinical Practices prior to the start of this study with periodic refresher training to ensure compliance with privacy principles and laws.

Risk of Discomfort: All participants will complete questionnaires at specified time points. Study participants in the intervention group will be asked to meet with a social worker at regularly scheduled clinic visits at month 2, 6, and 12. These questionnaires and discussions may be uncomfortable. Study staff trained in facilitation of questionnaires will conduct baseline interviews. Study staff will be trained with communication and interview skills to address and navigate potential stressors and discomfort. Social workers that conduct social work sessions will be trained in motivational interviewing and identifying referrals for mental health needs.

. In accordance with the informed consent, participants will be instructed that they are not required to disclose any personal or uncomfortable information and may withdraw from the study at any time.

As this study requires blood samples to be drawn, there is a risk of discomfort. There is a risk of mild pain, local irritation, bleeding or bruising at the puncture site. There is a small risk for light-headedness and/or fainting.

- 1. **Potential Benefits**

Patients in each of the four arms will receive enhanced care. The goal of this study is to improve adherence and long-term outcomes through differential service delivery. There is no guarantee of a direct benefit received by taking part in this study. However, the knowledge gained may guide investigators in improving treatment support and programs for individual patients in the current study and in the future.

- 1. **Alternatives**

Taking part in this study is voluntary. If potential participants decide not to take part in the study, he or she will not lose any of the regular benefits. Tuberculosis treatment is provided without cost in accordance with the South African TB Program (NTP) guidelines. If the participant decides not to continue this study, he or she may leave the study at any time without penalty. Leaving the study will not affect the standard of medical care.

- 1. **Adverse Events Reporting**

The risk of adverse events (AE) is low for participating in this study as there is no medicinal interventional in this study. However, as this study does include a behavioral intervention, AEs will be closely monitored.

An AE is defined as any untoward medical occurrence in a patient or clinical investigation subject administered a medicinal product and which does not necessarily have a causal relationship with the treatment. All adverse events will be documented and reported to the PI. Study related serious AEs will be reported to the proper Ethics Committee in accordance with regulations. The study team will discuss adverse events as they occur and steps to prevent recurrence.

An AE may include the loss of electronic data, paper-based data, or other potentially sensitive or identifying information. The Columbia University Medical Center Human Research Protection Office (HRPO) Institutional Review Boards (IRB) and BREC will be informed of potential privacy breaches in accordance with reporting guidelines.

**OVERALL STRUCTURE OF THE TEAM**

**
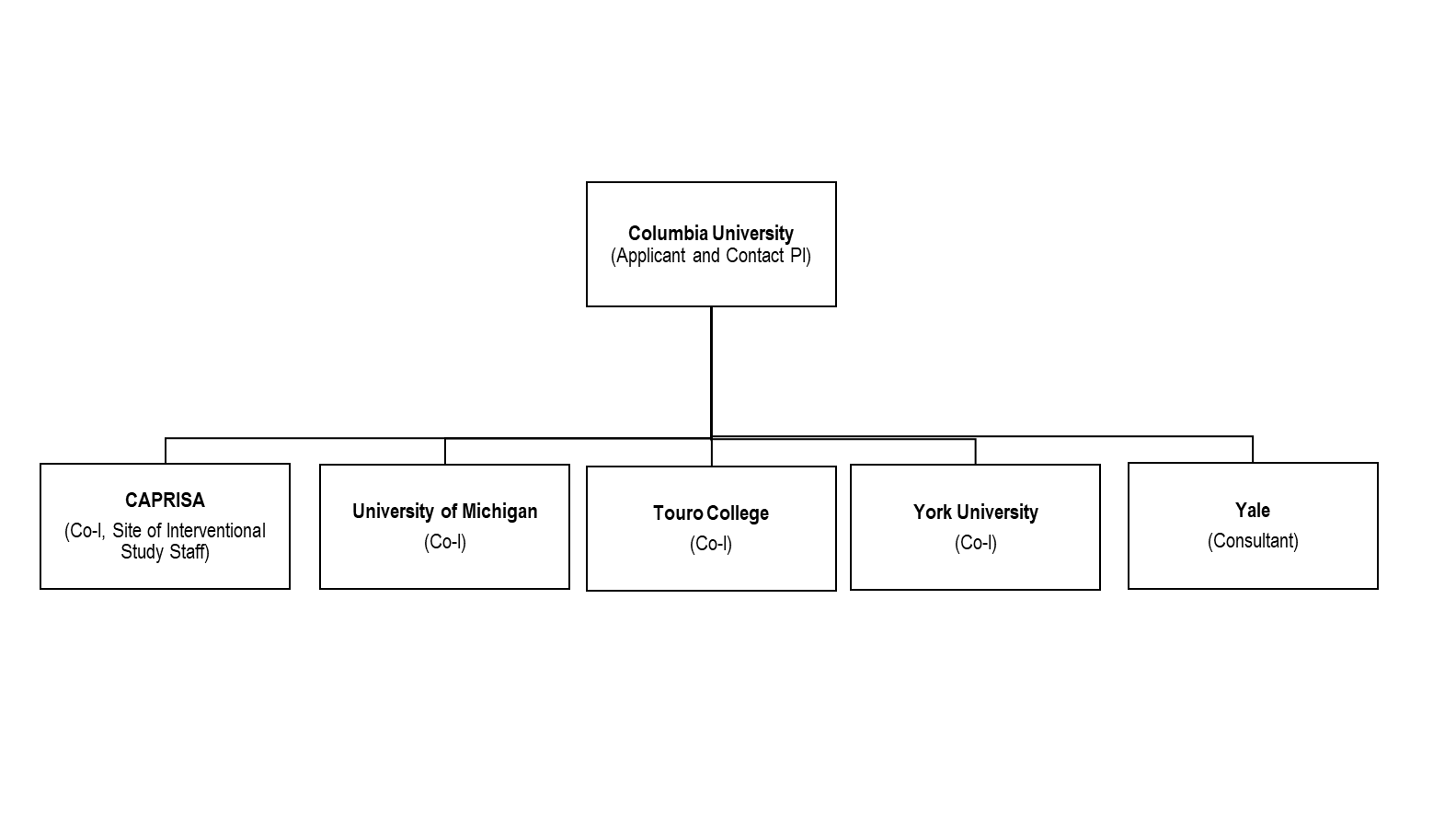
**

**Columbia University**: Columbia University will serve as the applicant and lead institution and data coordinating site in the collaboration. Dr. O'Donnell, who is also an Associate Research Scientist at CAPRISA, will oversee all aspects of the project.

Columbia University is the site of co-Investigator Professor Ying Kuen 'Ken' Cheung. He will be responsible for the development and implementation of the Bayesian adaptive study design and randomization software (Shiny App), which serves as the overall clinical structure for the project. Dr. Cheung will conduct training remotely for any members of the study team that are responsible for randomization and enrollment activities.

Study staff, including the PI at Columbia University will participate in staff trainings both remotely and in-person by traveling to South Africa. When not on-site, bi-weekly teleconferences will be conducted between Columbia University and the study staff at CAPRISA for oversight of the clinical operations of the study and to review data collection. The PI will ensure that regulatory documents are maintained and up-to-date. The site PIs will collaborate to devise standard operating procedure (SOP) documents that will be the basis for all training to ensure consistency. The Columbia University PI will travel to South Africa at least two times prior to study initiation for training and oversite.

Study staff at Columbia University will have access to the online study database and will oversee data collection and efforts to ensure timely and accurate data entry. When data is entered into the study database, it will be entered without direct identifiers. Study participants will be assigned a study ID, which will be used to enter data into study forms. Only the CAPRISA team will have access to the key.

Subcontracted sites: CAPRISA, Touro University, York University, University of Michigan, and Yale University will receive funding from the NIH R01 grant and have fully executed agreements in place. Columbia will be responsible for the overall administration of subcontracts for the institutions involved in this study.

**Centre for the AIDS Programme of Research in South Africa (CAPRISA)**: The site Principal Investigator, Dr. Kogieleum Naidoo will oversee the daily activities conducted at the clinical site in KwaZulu-Natal. She will supervise the team from enrollment through follow-up. **All patients will be enrolled by CAPRISA study staff at King DinuZulu Hospital and affiliated clinics**. The remaining sub-sites will be involved only in oversight, study design, data analysis, and manuscript preparation.

Site co-Investigator Dr. Boitumelo Seepamore is faculty in the Department of Social Work with expertise in medical social work, HIV/AIDS, counselling, and group support work. She brings unique skills as a fluent isiZulu speaker, and will provide nuance to training and interpretation of qualitative themes. She will supervise on the ground, and in person, sociobehavioral teams.

Study staff at CAPRISA will be responsible for timely and accurate data entry into the Shiny App to ensure appropriate randomization assignments for enrolled patients.

CAPRISA will be the site of the clinical study implementation and administration, which includes data collection and storage and maintenance. CAPRISA has a dedicated data management team that will be responsible for building and maintaining the study database. The database will be programed to scan for missing or out of range data. Designated personnel at both CAPRISA and Columbia University will have access to the study database and will regularly review the database for completeness. Study staff at CAPRISA will be responsible for entry of missing or erroneous data.

The study team will be responsible for recruitment and retention of patients. Dr. Naidoo will be responsible for the development of the study protocol through the course of the study. Dr. Naidoo will be responsible for the review of AEs and SAEs. The study team at CAPRISA will be responsible for documenting AEs/SAEs. The Columbia University PI will be made aware of AEs by the CAPRISA.

CAPRISA will have an onsite study coordinator that will oversee all regulatory requirements related to the study and will ensure that renewals will be submitted in a timely fashion. The coordinator will also ensure compliance with federal Clinical Trials guidance, including submission to Clinicaltrials.gov. The PIs at Columbia University and CAPRISA will work closely with the coordinator to ensure that regulatory aspects of the study are up-to-date and according to regulations. Regulatory documents will be shared with the Columbia study team via a secured Sharepoint folder such that the Columbia PI and Study Coordinator and review and ensure that documentation is current.

The on-site study team will oversee the randomization (minimization) process, supervise the socio-behavioral teams, and implement and supervise the mHealth intervention. The team at CAPRISA has extensive experience with Wisepill RT2000 devices including support maintenance and troubleshooting.

Study staff at CAPRISA will be responsible for collecting study biological samples (blood and sputum). Study staff will transport samples per protocol to the designated CAPRISA laboratory for processing and storage.

Ongoing monitoring/auditing of collected study data will be periodically performed at CAPRISA by CAPRISA personnel.

The **Biomedical Research Ethics Committee** (BREC) is responsible for evaluating and ethics approval to conduct this study in South Africa and covers both the work done at CAPRISA and King DinuZulu Hospital and affiliated clinics.

**Touro College Graduate School of Social Work**: Site co-Investigator Dr. Zelnick will oversee the social behavioral aspects of the intervention and staff, which include psychosocial support, specifically individual counselling sessions and adherence support groups. Her expertise is in psychosocial support for TB and HIV patients and has worked in South Africa for nearly 20 years. She will also supervise and train, providing career development support to early career co-Investigator, Dr. Boitumelo Seepamore. She will participate in both qualitative, quantitative analysis, and the preparation of manuscripts. Drs. Zelnick and Seepamore will lead the training of social workers and counselors to deliver the Enhanced Standard of Care and the social behavioral aspects of the intervention.

The purpose of the study is to react to barriers to adherence in a timely manner with referrals to proper resources. Drs. Zelnick and Seepamore will lead the training of social workers to support all enrolled patients as needed. The purpose of the depression screen is to identify at-risk patients in a timely manner and provide support and referrals. A full-time data manager will be also review all questionnaires and data collected on a daily basis and will support with identifying at-risk patients and notify the social support team.

**University of Michigan**: Site co-Investigator Dr. K. Rivet Amico has expertise in adherence, model-based psychosocial and behavioral interventions, and social science epidemiology. She will be involved in the development, training, and oversight of the social-behavioral aspects of the intervention. She will lead the analysis of quantitative social-behavioral data and will participate in manuscript preparation.

**York University**: Site co-Investigator Dr. Amrita Daftary is an experienced TB social scientist and qualitative researcher. She will be involved in training staff to deliver the social behavioral aspects of the intervention, develop interview guides and perform all qualitative analysis. She will lead preparation for qualitative manuscripts.

**Yale University**: Consultant Dr. Gerald Friedland will be involved in study design, interpretation of results and manuscript preparation. He will be involved in the design, training, implementation and evaluation of the adherence intervention.

**Data Safety and Monitoring Board**: The proposed study is a 4-armed adaptive randomized intervention. Within the context of the intervention, all medical care will be provided according to existing standards of care and practices. However, given the behavioral intervention, there is a small but real risk of harm to the individual and there is also a risk between groups (control versus intervention) of a lack of equipoise that could emerge during the course of the trial. For these reasons, we will organize a data safety and monitoring committee (DSMB) . After convening the DSMB, an initiation meeting will be scheduled to establish committee processes and familiarize members with study protocols. At four check points during the course of the study the DSMB will be presented with data regarding adverse events, mortality and study efficacy. This will occur after the first 40 patients have accrued 6-months of study time, and thereafter twice per year during years 1-4 of the study period. Additionally, data regarding unanticipated adverse events, mortality and protocol violations will be monitored in real-time, and reported to the relevant ethical/regulatory bodies throughout the study. The DSMB will have a final meeting at study close date (year 5) to review any late adverse events.

The DSMB could, in exceptional circumstances, recommend termination of the study or termination of one of the treatment regimens due to unacceptable levels of adverse events, or mortality; the trial should not be modified on account of differences in efficacy between treatment arms unless there is a concern for patient safety. Since the trial interventions are adherence support modalities study termination due to excess adverse events is unlikely. The DSMB will be asked to give advice on whether the accumulated data from the trial, together with results from other relevant trials, justifies continuing recruitment of further patients. They may recommend modification or closure of the study.

1. REFERENCES

1. Diacon AH, Pym A, Grobusch MP, de los Rios JM, Gotuzzo E, Vasilyeva I, et al. Multidrug-resistant tuberculosis and culture conversion with bedaquiline. N Engl J Med. 2014;371(8):723-32.

2. O'Donnell MR, Padayatchi N, Daftary A, Orrell C, Dooley KE, Rivet Amico K, et al. Antiretroviral switching and bedaquiline treatment of drug-resistant tuberculosis HIV co-infection. Lancet HIV. 2019;6(3):e201-e4.

3. Calmy A, Tovar Sanchez T, Kouanfack C, Mpoudi-Etame M, Leroy S, Perrineau S, et al. Dolutegravir-based and low-dose efavirenz-based regimen for the initial treatment of HIV-1 infection (NAMSAL): week 96 results from a two-group, multicentre, randomised, open label, phase 3 non-inferiority trial in Cameroon. Lancet HIV. 2020;7(10):e677-e87.

4. Group NAS, Kouanfack C, Mpoudi-Etame M, Omgba Bassega P, Eymard-Duvernay S, Leroy S, et al. Dolutegravir-Based or Low-Dose Efavirenz-Based Regimen for the Treatment of HIV-1. N Engl J Med. 2019;381(9):816-26.

5. Nimmo C, Millard J, Brien K, Moodley S, van Dorp L, Lutchminarain K, et al. Bedaquiline resistance in drug-resistant tuberculosis HIV co-infected patients. Eur Respir J. 2020;55(6).

6. Nimmo C, Millard J, van Dorp L, Brien K, Moodley S, Wolf A, et al. Population-level emergence of bedaquiline and clofazimine resistance-associated variants among patients with drug-resistant tuberculosis in southern Africa: a phenotypic and phylogenetic analysis. Lancet Microbe. 2020;1(4):e165-e74.

7. WHO. World Health Organization. Global tuberculosis report 2020. 2020.

8. Saunders MJ, Evans CA. COVID-19, tuberculosis and poverty: preventing a perfect storm. Eur Respir J. 2020;56(1).

9. Gupta RK, Lucas SB, Fielding KL, Lawn SD. Prevalence of tuberculosis in post-mortem studies of HIV-infected adults and children in resource-limited settings: a systematic review and meta-analysis. AIDS. 2015;29(15):1987-2002.

10. Zellweger JP, Heinzer R, Touray M, Vidondo B, Altpeter E. Intra-observer and overall agreement in the radiological assessment of tuberculosis. Int J Tuberc Lung Dis. 2006;10(10):1123-6.

11. WHO. World Health Organization. The End TB Strategy. 2015. [Available from: <https://www.who.int/tb/post2015_strategy/en/>.

12. Mabhula A, Singh V. Drug-resistance in Mycobacterium tuberculosis: where we stand. Medchemcomm. 2019;10(8):1342-60.

13. Ahuja SD, Ashkin D, Avendano M, Banerjee R, Bauer M, Bayona JN, et al. Multidrug resistant pulmonary tuberculosis treatment regimens and patient outcomes: an individual patient data meta-analysis of 9,153 patients. PLoS Med. 2012;9(8):e1001300.

14. Cohen T, Murray M, Wallengren K, Alvarez GG, Samuel EY, Wilson D. The prevalence and drug sensitivity of tuberculosis among patients dying in hospital in KwaZulu-Natal, South Africa: a postmortem study. PLoS Med. 2010;7(6):e1000296.

15. Abdool Karim Q, Abdool Karim SS. COVID-19 affects HIV and tuberculosis care. Science. 2020;369(6502):366.

16. Basu S, Andrews JR, Poolman EM, Gandhi NR, Shah NS, Moll A, et al. Prevention of nosocomial transmission of extensively drug-resistant tuberculosis in rural South African district hospitals: an epidemiological modelling study. Lancet. 2007;370(9597):1500-7.

17. Shah NS, Auld SC, Brust JC, Mathema B, Ismail N, Moodley P, et al. Transmission of Extensively Drug-Resistant Tuberculosis in South Africa. N Engl J Med. 2017;376(3):243-53.

18. Adane AA, Alene KA, Koye DN, Zeleke BM. Non-adherence to anti-tuberculosis treatment and determinant factors among patients with tuberculosis in northwest Ethiopia. PloS one. 2013;8(11):e78791.

19. Kulkarni P, Akarte S, Mankeshwar R, Bhawalkar J, Banerjee A, Kulkarni A. Non-adherence of new pulmonary tuberculosis patients to anti-tuberculosis treatment. Annals of medical and health sciences research. 2013;3(1):67-74.

20. Gandhi NR, Moll A, Sturm AW, Pawinski R, Govender T, Lalloo U, et al. Extensively drug-resistant tuberculosis as a cause of death in patients co-infected with tuberculosis and HIV in a rural area of South Africa. Lancet. 2006;368(9547):1575-80.

21. Gardner EM, McLees MP, Steiner JF, Del Rio C, Burman WJ. The spectrum of engagement in HIV care and its relevance to test-and-treat strategies for prevention of HIV infection. Clin Infect Dis. 2011;52(6):793-800.

22. Senthilingam M, Pietersen E, McNerney R, Te Riele J, Sedres P, Wilson R, et al. Lifestyle, attitudes and needs of uncured XDR-TB patients living in the communities of South Africa: a qualitative study. Trop Med Int Health. 2015.

23. Daftary A, Padayatchi N, O'Donnell M. Preferential adherence to antiretroviral therapy over tuberculosis treatment: a qualitative study of drug-resistant TB/HIV co-infected patients in South Africa. Glob Public Health. 2014;9(9):1107-16.

24. Daftary A, Padayatchi N. Social constraints to TB/HIV healthcare: accounts from coinfected patients in South Africa. AIDS care. 2012;24(12):1480-6.

25. NIAID Research Agenda Multidrug-Resistant and Extensively Drug-Resistant Tuberculosis. NIAID. 2007.

26. Mansoor LE, Karim QA, Werner L, Madlala B, Ngcobo N, Cornman DH, et al. Impact of an adherence intervention on the effectiveness of tenofovir gel in the CAPRISA 004 trial. AIDS Behav. 2014;18(5):841-8.

27. O'Laughlin KN, Wyatt MA, Kaaya S, Bangsberg DR, Ware NC. How treatment partners help: social analysis of an African adherence support intervention. AIDS Behav. 2012;16(5):1308-15.

28. Brust JC, Gandhi NR, Carrara H, Osburn G, Padayatchi N. High treatment failure and default rates for patients with multidrug-resistant tuberculosis in KwaZulu-Natal, South Africa, 2000-2003. Int J Tuberc Lung Dis. 2010;14(4):413-9.

29. Nelson KN, Shah NS, Mathema B, Ismail N, Brust JCM, Brown TS, et al. Spatial Patterns of Extensively Drug-Resistant Tuberculosis Transmission in KwaZulu-Natal, South Africa. J Infect Dis. 2018;218(12):1964-73.

30. Brown TS, Challagundla L, Baugh EH, Omar SV, Mustaev A, Auld SC, et al. Pre-detection history of extensively drug-resistant tuberculosis in KwaZulu-Natal, South Africa. Proc Natl Acad Sci U S A. 2019;116(46):23284-91.

31. Isaakidis P, Casas EC, Das M, Tseretopoulou X, Ntzani EE, Ford N. Treatment outcomes for HIV and MDR-TB co-infected adults and children: systematic review and meta-analysis. Int J Tuberc Lung Dis. 2015;19(8):969-78.

32. Singh A, Prasad R, Balasubramanian V, Gupta N. Drug-Resistant Tuberculosis and HIV Infection: Current Perspectives. HIV AIDS (Auckl). 2020;12:9-31.

33. Walmsley SL, Antela A, Clumeck N, Duiculescu D, Eberhard A, Gutierrez F, et al. Dolutegravir plus abacavir-lamivudine for the treatment of HIV-1 infection. N Engl J Med. 2013;369(19):1807-18.

34. Vitoria M, Hill A, Ford N, Doherty M, Clayden P, Venter F, et al. The transition to dolutegravir and other new antiretrovirals in low-income and middle-income countries: what are the issues? AIDS. 2018;32(12):1551-61.

35. Schnippel K, Ndjeka N, Maartens G, Meintjes G, Master I, Ismail N, et al. Effect of bedaquiline on mortality in South African patients with drug-resistant tuberculosis: a retrospective cohort study. Lancet Respir Med. 2018;6(9):699-706.

36. Meireles MV, Pascom ARP, Duarte EC, McFarland W. Comparative effectiveness of first-line antiretroviral therapy: results from a large real-world cohort after the implementation of dolutegravir. AIDS. 2019;33(10):1663-8.

37. Howell EM, Kigozi NG, Heunis JC. Community-based directly observed treatment for TB patients to improve HIV services: a cross-sectional study in a South African province. BMC Health Serv Res. 2018;18(1):255.

38. Mannheimer S, Friedland G, Matts J, Child C, Chesney M. The consistency of adherence to antiretroviral therapy predicts biologic outcomes for human immunodeficiency virus-infected persons in clinical trials. Clin Infect Dis. 2002;34(8):1115-21.

39. Alipanah N, Jarlsberg L, Miller C, Linh NN, Falzon D, Jaramillo E, et al. Adherence interventions and outcomes of tuberculosis treatment: A systematic review and meta-analysis of trials and observational studies. PLoS Med. 2018;15(7):e1002595.

40. Iacob SA, Iacob DG, Jugulete G. Improving the Adherence to Antiretroviral Therapy, a Difficult but Essential Task for a Successful HIV Treatment-Clinical Points of View and Practical Considerations. Front Pharmacol. 2017;8:831.

41. van den Boogaard J, Boeree MJ, Kibiki GS, Aarnoutse RE. The complexity of the adherence-response relationship in tuberculosis treatment: why are we still in the dark and how can we get out? Trop Med Int Health. 2011;16(6):693-8.

42. O'Donnell MR, Daftary A, Frick M, Hirsch-Moverman Y, Amico KR, Senthilingam M, et al. Re-inventing adherence: toward a patient-centered model of care for drug-resistant tuberculosis and HIV. Int J Tuberc Lung Dis. 2016;20(4):430-4.

43. Stephens F, Gandhi NR, Brust JCM, Mlisana K, Moodley P, Allana S, et al. Treatment Adherence Among Persons Receiving Concurrent Multidrug-Resistant Tuberculosis and HIV Treatment in KwaZulu-Natal, South Africa. J Acquir Immune Defic Syndr. 2019;82(2):124-30.

44. O'Donnell MR, Wolf A, Werner L, Horsburgh CR, Padayatchi N. Adherence in the treatment of patients with extensively drug-resistant tuberculosis and HIV in South Africa: a prospective cohort study. J Acquir Immune Defic Syndr. 2014;67(1):22-9.

45. Daftary A, Mondal S, Zelnick JR, Friedland G, Seepamore B, Boodhram R, et al. Dynamic Needs and Challenges of People with Drug-Resistant Tuberculosis and HIV in South Africa: A Qualitative Study. Lancet. 2020;Ahead of Print.

46. Law S, Daftary A, O'Donnell M, Padayatchi N, Calzavara L, Menzies D. Interventions to improve retention-in-care and treatment adherence among patients with drug-resistant tuberculosis: a systematic review. Eur Respir J. 2019;53(1).

47. Companion Handbook to the WHO Guidelines for the Programmatic Management of Drug-Resistant Tuberculosis. WHO Guidelines Approved by the Guidelines Review Committee. Geneva2014.

48. Bionghi N, Daftary A, Maharaj B, Msibi Z, Amico KR, Friedland G, et al. Pilot evaluation of a second-generation electronic pill box for adherence to Bedaquiline and antiretroviral therapy in drug-resistant TB/HIV co-infected patients in KwaZulu-Natal, South Africa. BMC Infect Dis. 2018;18(1):171.

49. Zelnick JR, Daftary A, Hwang C, Labar AS, Boodhram R, Maharaj B, et al. Electronic dose monitoring identifies a high-risk subpopulation in the treatment of drug-resistant tuberculosis and HIV. Clin Infect Dis. 2020.

50. MacCarthy S, Mendoza-Graf A, Saya U, Samba C, Birungi J, Okoboi S, et al. Lessons learned from a mobile technology-based intervention informed by behavioral economics to improve ART adherence among youth in Uganda. AIDS care. 2019:1-7.

51. Haberer JE, Sabin L, Amico KR, Orrell C, Galarraga O, Tsai AC, et al. Improving antiretroviral therapy adherence in resource-limited settings at scale: a discussion of interventions and recommendations. J Int AIDS Soc. 2017;20(1):21371.

52. Rogers BG, Lee JS, Safren SA. Behavioral Interventions for Adherence. In: Hope TJ, Richman D, Stevenson M, editors. Encyclopedia of AIDS. New York, NY: Springer New York; 2017. p. 1-6.

53. Shaw S, Amico KR. Antiretroviral Therapy Adherence Enhancing Interventions for Adolescents and Young Adults 13-24 Years of Age: A Review of the Evidence Base. J Acquir Immune Defic Syndr. 2016;72(4):387-99.

54. Cooper V, Clatworthy J, Whetham J, Consortium E. mHealth Interventions To Support Self-Management In HIV: A Systematic Review. Open AIDS J. 2017;11:119-32.

55. Chiang N, Guo M, Amico KR, Atkins L, Lester RT. Interactive Two-Way mHealth Interventions for Improving Medication Adherence: An Evaluation Using The Behaviour Change Wheel Framework. JMIR Mhealth Uhealth. 2018;6(4):e87.

56. Haldane V, Koh JJK, Srivastava A, Teo KWQ, Tan YG, Cheng RX, et al. User Preferences and Persona Design for an mHealth Intervention to Support Adherence to Cardiovascular Disease Medication in Singapore: A Multi-Method Study. JMIR Mhealth Uhealth. 2019;7(5):e10465.

57. Brewer LC, Jenkins S, Lackore K, Johnson J, Jones C, Cooper LA, et al. mHealth Intervention Promoting Cardiovascular Health Among African-Americans: Recruitment and Baseline Characteristics of a Pilot Study. JMIR Res Protoc. 2018;7(1):e31.

58. Zhuang Q, Chen F, Wang T. Effectiveness of short message service intervention to improve glycated hemoglobin control and medication adherence in type-2 diabetes: A meta-analysis of prospective studies. Prim Care Diabetes. 2019.

59. Menon V, Selvakumar N, Kattimani S, Andrade C. Therapeutic effects of mobile-based text message reminders for medication adherence in bipolar I disorder: Are they maintained after intervention cessation? J Psychiatr Res. 2018;104:163-8.

60. Xiao Q, Wang J, Chiang V, Choi T, Wang Y, Sun L, et al. Effectiveness of mHealth Interventions for Asthma Self-Management: A Systematic Review and Meta-Analysis. Stud Health Technol Inform. 2018;250:144-5.

61. Schindler-Ruwisch JM, Roess A, Robert RC, Napolitano MA, Chiang S. Social Support for Breastfeeding in the Era of mHealth: A Content Analysis. J Hum Lact. 2018;34(3):543-55.

62. Zelnick JR, Seepamore B, Daftary A, Amico KR, Bhengu X, Friedland G, et al. Training social workers to enhance patient-centered care for drug-resistant TB-HIV in South Africa. Public Health Action. 2018;8(1):25-7.

63. Rabkin M, Howard AA, Ehrenkranz P, Fernandez LG, Preko P, Singh V, et al. Leveraging differentiated HIV service delivery to expand tuberculosis preventive treatment: a call to action. Int J Tuberc Lung Dis. 2020;24(2):165-9.

64. Ehrenkranz P, Grimsrud A, Holmes CB, Preko P, Rabkin M. Expanding the vision for differentiated service delivery: a call for more inclusive and truly patient-centered care for people living with HIV. J Acquir Immune Defic Syndr. 2020.

65. Ehrenkranz P, Grimsrud A, Rabkin M. Differentiated service delivery: navigating the path to scale. Curr Opin HIV AIDS. 2019;14(1):60-5.

66. Roy M, Bolton Moore C, Sikazwe I, Holmes CB. A Review of Differentiated Service Delivery for HIV Treatment: Effectiveness, Mechanisms, Targeting, and Scale. Curr HIV/AIDS Rep. 2019;16(4):324-34.

67. E. G. Stigma: notes on the management of spoiled identity. USA: Simons & Schuster, Inc.; 1963.

68. Pescosolido BA, Martin JK. The Stigma Complex. Annual review of sociology. 2015;41:87-116.

69. Parker R, Aggleton P. HIV and AIDS-related stigma and discrimination: a conceptual framework and implications for action. Soc Sci Med. 2003;57(1):13-24.

70. Stangl AL, Lloyd JK, Brady LM, Holland CE, Baral S. A systematic review of interventions to reduce HIV-related stigma and discrimination from 2002 to 2013: how far have we come? Journal of the International AIDS Society. 2013;16(3Suppl 2):18734.

71. Hirsch-Moverman Y, Daftary A, Yuengling KA, Saito S, Ntoane M, Frederix K, et al. Using mHealth for HIV/TB Treatment Support in Lesotho: Enhancing Patient-Provider Communication in the START Study. J Acquir Immune Defic Syndr. 2017;74 Suppl 1(Suppl 1):S37-s43.

72. Edginton ME, Sekatane CS, Goldstein SJ. Patients' beliefs: do they affect tuberculosis control? A study in a rural district of South Africa. Int J Tuberc Lung Dis. 2002;6(12):1075-82.

73. Moller V, Erstad I. Stigma asociated with tuberculosis in a time of HIV/AIDS: narratives from the Eastern Cape, South Africa. South African Review of Sociology. 2007;38(2):103-19.

74. Daftary A. HIV and tuberculosis: the construction and management of double stigma. Soc Sci Med. 2012;74(10):1512-9.

75. Nyblade LC. Measuring HIV stigma: existing knowledge and gaps. Psychol Health Med. 2006;11(3):335-45.

76. Daftary A, Mondal S, Zelnick J, Friedland G, Seepamore B, Boodhram R, et al. Dynamic needs and challenges of people with drug-resistant tuberculosis and HIV in South Africa: a qualitative study. Lancet Glob Health. 2021;9(4):e479-e88.

77. Gao J, Su PF, Hu F, Cheung SH. Adaptive treatment allocation for comparative clinical studies with recurrent events data. Biometrics. 2019.

78. Mansoor LE, Abdool Karim Q, Mngadi KT, Dlamini S, Montague C, Nkomonde N, et al. Assessing the implementation effectiveness and safety of 1% tenofovir gel provision through family planning services in KwaZulu-Natal, South Africa: study protocol for an open-label randomized controlled trial. Trials. 2014;15:496.

79. Lassi ZS, Kumar R, Mansoor T, Salam RA, Das JK, Bhutta ZA. Essential interventions: implementation strategies and proposed packages of care. Reprod Health. 2014;11 Suppl 1:S5.

80. THOMPSON WR. ON THE LIKELIHOOD THAT ONE UNKNOWN PROBABILITY EXCEEDS ANOTHER IN VIEW OF THE EVIDENCE OF TWO SAMPLES. Biometrika. 1933;25(3-4):285-94.

81. Cheung YK, Inoue LY, Wathen JK, Thall PF. Continuous Bayesian adaptive randomization based on event times with covariates. Stat Med. 2006;25(1):55-70.

82. Cheung YK, Thall PF. Monitoring the rates of composite events with censored data in phase II clinical trials. Biometrics. 2002;58(1):89-97.

83. Miller WR, Moyers TB. Motivational interviewing and the clinical science of Carl Rogers. J Consult Clin Psychol. 2017;85(8):757-66.

84. Yuengling KA, Padayatchi N, Wolf A, Mathema B, Brown T, Horsburgh CR, et al. Effect of Antiretroviral Therapy on Treatment Outcomes in a Prospective Study of Extensively Drug-Resistant Tuberculosis (XDR-TB) HIV Coinfection Treatment in KwaZulu-Natal, South Africa. J Acquir Immune Defic Syndr. 2018;79(4):474-80.

85. Visser M, Mundell J, de Villiers A, Sikkema K, Jeffery B. Development of structured support groups for HIV-positive women in South Africa. Sahara j. 2005;2(3):333-43.

86. Lennon-Dearing R. The Benefits of Women-Only HIV Support Groups. Journal of HIV/AIDS & Social Services. 2008;7(1):27-45.

87. Murray SA, Kendall M, Carduff E, Worth A, Harris FM, Lloyd A, et al. Use of serial qualitative interviews to understand patients' evolving experiences and needs. BMJ. 2009;339:b3702.

88. Horby PW, Estcourt L, Peto L, Emberson JR, Staplin N, Spata E, et al. Convalescent plasma in patients admitted to hospital with COVID-19 (RECOVERY): a randomised, controlled, open-label, platform trial. medRxiv. 2021:2021.03.09.21252736.
